# Food additives: distribution and co-occurrence in 126,000 food products of the French market

**DOI:** 10.1038/s41598-020-60948-w

**Published:** 2020-03-04

**Authors:** Eloi Chazelas, Mélanie Deschasaux, Bernard Srour, Emmanuelle Kesse-Guyot, Chantal Julia, Benjamin Alles, Nathalie Druesne-Pecollo, Pilar Galan, Serge Hercberg, Paule Latino-Martel, Younes Esseddik, Fabien Szabo, Pierre Slamich, Stephane Gigandet, Mathilde Touvier

**Affiliations:** 1 Sorbonne Paris Nord - Paris 13 University, Inserm U1153, Inrae U1125, Cnam, Nutritional Epidemiology Research Team (EREN), Epidemiology and Statistics Research Center – University of Paris (CRESS), Bobigny, France; 2 Public Health Department, Avicenne Hospital, AP-HP, Bobigny, France; 3 Open Food Facts, Saint-Maur-des-Fossés, France

**Keywords:** Diseases, Health care, Medical research, Risk factors

## Abstract

Background. More than 330 food additives (e.g. artificial sweeteners, emulsifiers, dyes) are authorized in Europe, with a great variability of use across food products. Objective. The objective of this study was to investigate the distribution and co-occurrence of food additives in a large-scale database of foods and beverages available on the French market. Design. The open access crowdsourced Open Food Facts database (https://world.openfoodfacts.org/) was used to retrieve the composition of food and beverage products commonly marketed on the French market (n = 126,556), based on the ingredients list. Clustering of food additive variables was used in order to determine groups of additives frequently co-occurring in food products. The clusters were confirmed by network analysis, using the *eLasso* method. Results. Fifty-three-point eight percent of food products contained at least 1 food additive and 11.3% at least 5. Food categories most likely to contain food additives (in more than 85% of food items) were artificially sweetened beverages, ice creams, industrial sandwiches, biscuits and cakes. The most frequently used food additives were citric acid, lecithins and modified starches (>10,000 products each). Some food additives with suspected health effects also pertained to the top 50: sodium nitrite, potassium nitrate, carrageenan, monosodium glutamate, sulfite ammonia caramel, acesulfame K, sucralose, (di/tri/poly) phosphates, mono- and diglycerides of fatty acids, potassium sorbate, cochineal, potassium metabisulphite, sodium alginate, and bixin (>800 food products each). We identified 6 clusters of food additives frequently co-occurring in food products. Conclusions. Food additives are widespread in industrial French products and some clusters of additives frequently co-occurring in food products were identified. These results pave the way to future etiological studies merging composition data to food consumption data to investigate their association with chronic disease risk, in particular potential ‘cocktail effects’.

## Introduction

In the Western world, the last decades were marked by an increase in the consumption of ‘ultra-processed’ foods, i.e. foods undergoing multiple physical, biological, and/or chemical processes and containing various food additives^1–6^. In France, the 2017 nationally representative INCA3 nutritional survey highlighted a rise in processed food consumption, mainly accounted for by industrially-processed foods^7^. In this context, results of observational epidemiological studies linking ‘ultra-processed’ food intake and health outcomes is accumulating worldwide^8–17^. A clinical trial also highlighted an association between ultra-processed food and increased ad libitum energy intake and weight gain over a 2-week period^18^. In addition to the poorer nutritional composition, the presence of neo-formed compounds and of substances migrating from packaging, food additives is one of the main hypotheses that could help explain these results. Ultra-processed foods indeed often contain mixtures of additives. They represent about 330 authorized compounds in the European Union (EU)^19^, where their use is legislated by Regulation (EC) No 1333/2008. The restriction may vary, for example, only 48 food additives are allowed in organic food products. The additive content of a food product is mandatorily provided on its packaging/label with a list of all substances identified by their E number (EU identifier), name (e.g. E466 carboxymethylcellulose) and function in the final product. They are commonly used as antioxidants, dyes, emulsifiers, stabilizers, gelling agents, thickeners, preservatives and sweeteners^20^, and some are undoubtedly useful for increasing shelf life and food safety. Most of them probably have no impact on health and some may even be beneficial (e.g. anti-microbial, antioxidants, polyphenols). However, some concerning results, mainly derived from animal and/or cell-based experimental studies, have emerged regarding several additives. For instance, nitrates/nitrites^21–23^, carrageenans^24^, glutamate^25–27^, bixin^28,29^, artificial sweeteners^30–34^, phosphates^35,36^, emulsifiers^37–39^, caramel^40,41^, titanium dioxide (TiO2)^42^, tartrazine^43,44^ and butylated hydroxyanisole/butylated hydroxytoluene (BHA/BHT)^43^ were previously linked to metabolic, gut microbiota or endocrine perturbations along with carcinogenic, inflammatory and/or oxidative stress effects. Besides, some experimental results suggest that different additives may interact (among themselves and/or with the food matrix) and thus lead to synergistic or antagonist effects, but few studies have been performed on this topic to-date^45–50^.

Maximum authorized levels of food additives are set by the European Food Safety Authority (EFSA)^20^ - and the WHO-FAO JECFA at the international level^51^ and are theoretically intended to protect consumers against the potential adverse effects of each individual substance in a given food product. Yet, despite the substantial amount of work on the literature review and the collective expertise at these institutions, the evaluation (and subsequent recommendations and regulations) has been based only on the currently available scientific evidence which is mainly derived from *in-vitro* or *in-vivo* experimental research and simulations of exposure in humans. In that context, information regarding: (1) the health impact of regular and cumulative intake of food additives in humans, and (2) the potential ‘cocktail’ effects/interactions is still missing yet urgently needed.

Furthermore, the presence of these substances in the foods available on the French market has been poorly studied. In order to pave the way for etiological studies, it is essential to document which food additives are the most widespread and in which food categories they are more likely to be found. In addition, the study of their co-occurrence in foods will identify various food additive mixtures that are relevant in real life. Thus, objectives of this work were (1) to investigate the distribution of food additives in a large-scale database of food and beverage products available on the French market and (2) to identify mixtures of food additives frequently co-occurring in food products raising the issue of possible cocktail effects.

## Methods

### Open food facts database

The Open Food Facts database was used to retrieve composition of food products (http://world.openfoodfacts.org/). Open Food Facts is an open collaborative database of food products marketed worldwide, licensed under the Open Database License (ODBL). This French initiative contains data on hundreds of thousands of products. The initiative started in France in 2012, providing extensive coverage of the French food market, and an increasing number of products are becoming available for other countries worldwide. Contributors (citizens and active Open Food Facts contributors) permanently add products to this crowdsourced database, by scanning the barcode and sending photographs of the packaging. The information is automatically treated to record a wealth of information for each food product, such as commercial name, brand, list of ingredients, presence/absence of each food additive and nutritional composition. As food products formulations may evolve, old products are regularly updated when they are re-informed by consumers.

The Global Trade Item Number (GTIN) embedded in the barcode acts as an identifier of each product.

For the present study, data was retrieved from the Open Food Facts database on April 10, 2019. Duplicates (different formats, e.g. packs “x4” or “x8”) were removed for products of the same brand and same composition. The information of additives in the list of ingredients is mandatory in Europe. All products currently marketed in France with available list of ingredients were included (n = 126,556, see Appendix 1 for flowchart) and corresponding information on the presence and nature of food additives was extracted for each food or beverage item. Food categorization has been previously described^52^. Thirty-five food categories were identified (Appendix 2).

### Distribution of food additives in foods and beverages

We calculated the percentage of food items in each food category, the percentage of food items containing at least one, two, three, etc. food additives, the percentage of food items containing at least one additive per food category, and the number of food products containing each food additive.

### Clustering of food additives frequently co-occurring in food products

In order to assess their co-occurrence in food and beverage items, clustering of food additives was performed using the R package ClustOfVar, specifically dedicated to the clustering of variables^53^. Each food product (n = 126,556) was described by presence/absence of each food additive (141 binary variables, after exclusion of food additives present in less than 100/126,556 food products). Following the ClustOfVar methodology, ascendant hierarchical clustering of variables was performed on this dataset (141 binary variables)^54^, thus providing clusters of food additives strongly co-occurring.

For each cluster, a squared loading is attributed to each additive, corresponding to the strength of the correlation between the food additive and its cluster. For each cluster of food additives variables, a synthetic variable (or score) is also generated by the package. For a given food product, the value of this synthetic variable for cluster_i_ increases when the number of food additives of cluster_i_ present in this food increases. In other words, the higher the number of cluster_i_ food additives in a given product, the higher the cluster_i_ synthetic variable for that product. For each cluster, food products with higher synthetic variable (>99th percentile of the distribution) were highlighted to show the food items which were the largest food additive carriers of this cluster. See Appendix 3 for more details on the ClustOfVar algorithm.

### Network analysis

In order to visualize the co-occurrence of food additives and to confirm the information provided by the clustering of variables by a complementary method, network analysis was performed with the R package IsingFit (see Appendix 4 for details). Based on the *eLasso* method, this package is specifically dedicated to the estimation of binary data network structures and provides an overview of the co-occurrences of additives, mutually adjusted for all the relationships of the network^55^. It can be interpreted as follows: when two food additives are connected by a blue line, it means that they are often found together in food products, when they are connected by a red line, it means that they are rarely found together. Bolder is the line, higher is the number of products concerned. For better visualization, the network was generated for the 50 food additives most present in food products, and additives were colored according to clusters defined by the ClustOfVar method.

### Additional descriptors

The Nutri-Score was used to provide information on the global nutritional quality of food products^56^. This score is based on a modified version of the Food Standard Agency (FSA) nutrient profiling system, and it has been endorsed by the French, Spanish, and Belgian governments as the official front-of-pack label in these countries (Appendix 5). It classifies foods into 5 classes, ranging from A/dark green (better nutritional quality) to E/dark orange-red (worst nutritional quality)^52,57^. Food products from the Open Food Facts database were also categorized into one of the four food NOVA groups, a food classification system developed by researchers from the University of São Paulo^58^, based on the extent and purpose of industrial food processing^58–60^. NOVA classifies all foods and food products into four groups: (1) unprocessed or minimally processed foods, (2) processed culinary ingredients, (3) processed foods, (4) ultra-processed foods (Appendix 6)^58^.

### Transparency statement

Dr. Touvier (the guarantor) affirms that the manuscript is an honest, accurate, and transparent account of the study being reported; that no important aspects of the study have been omitted; and that any discrepancies from the study as planned have been explained.

## Results

### Distribution of food items across food categories

**Figure 1:**
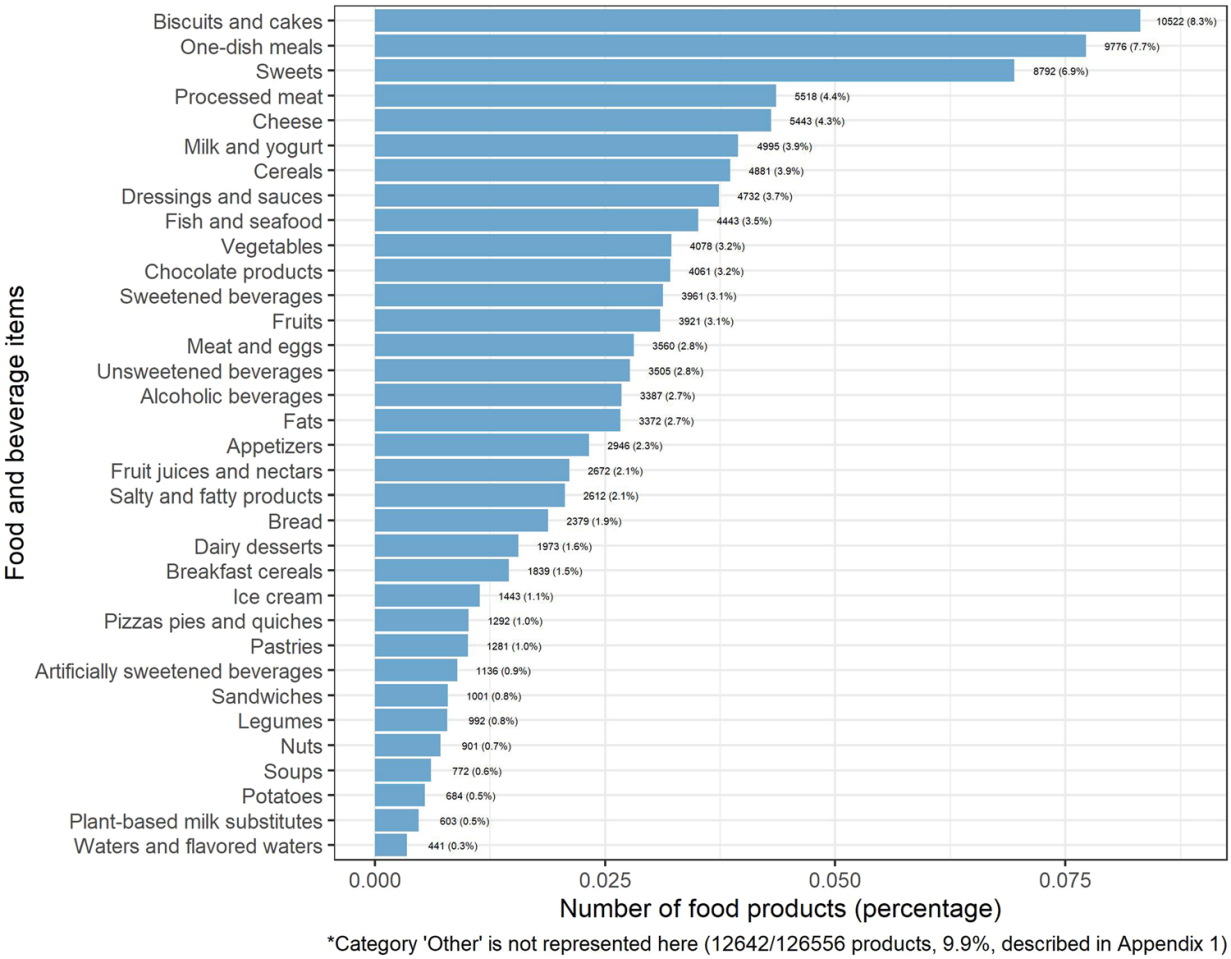
Percentage of food and beverage items by food category in the Open Food Facts database (n = 126,556 products), France 2019*.

The number and percentage of food or beverage items by food categories available in the Open Food Facts database is illustrated in Fig. 1. Out of 126,556 products, the most represented food categories were biscuits and cakes (8.3%), one-dish meals (7.7%), sweets (6.9%), processed meat (4.4%), cheese (4.3%), milk and yogurt (3.9%), cereal products (3.9%) and dressings and sauces (3.7%).

### Number of food additives per food product

**Figure 2:**
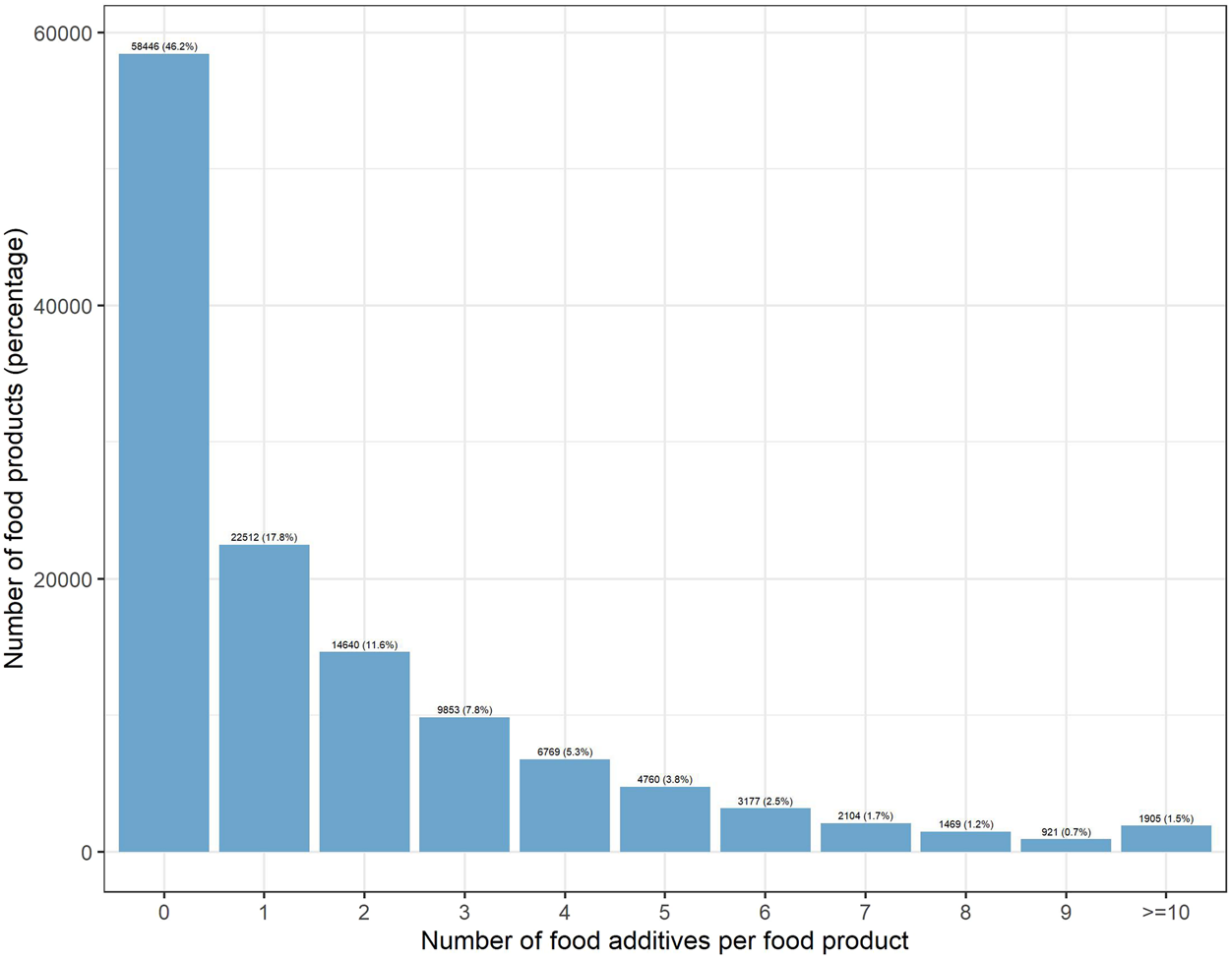
Number of food additives per food product, Open Food Facts database (n = 126,556 products), France 2019.

In all, 329 additives were found in the database, among which 141 were present in at least 100 food products. Figure 2 shows the number of food additives present in food products: overall 53.8% of products (68 110/126,556) contained at least one food additive; 17.8% contained one, 11.6% two, 7.8% three, 5.3% four and 11.3% five or more food additives.

### Proportion of food products containing additives in each food category

**Figure 3:**
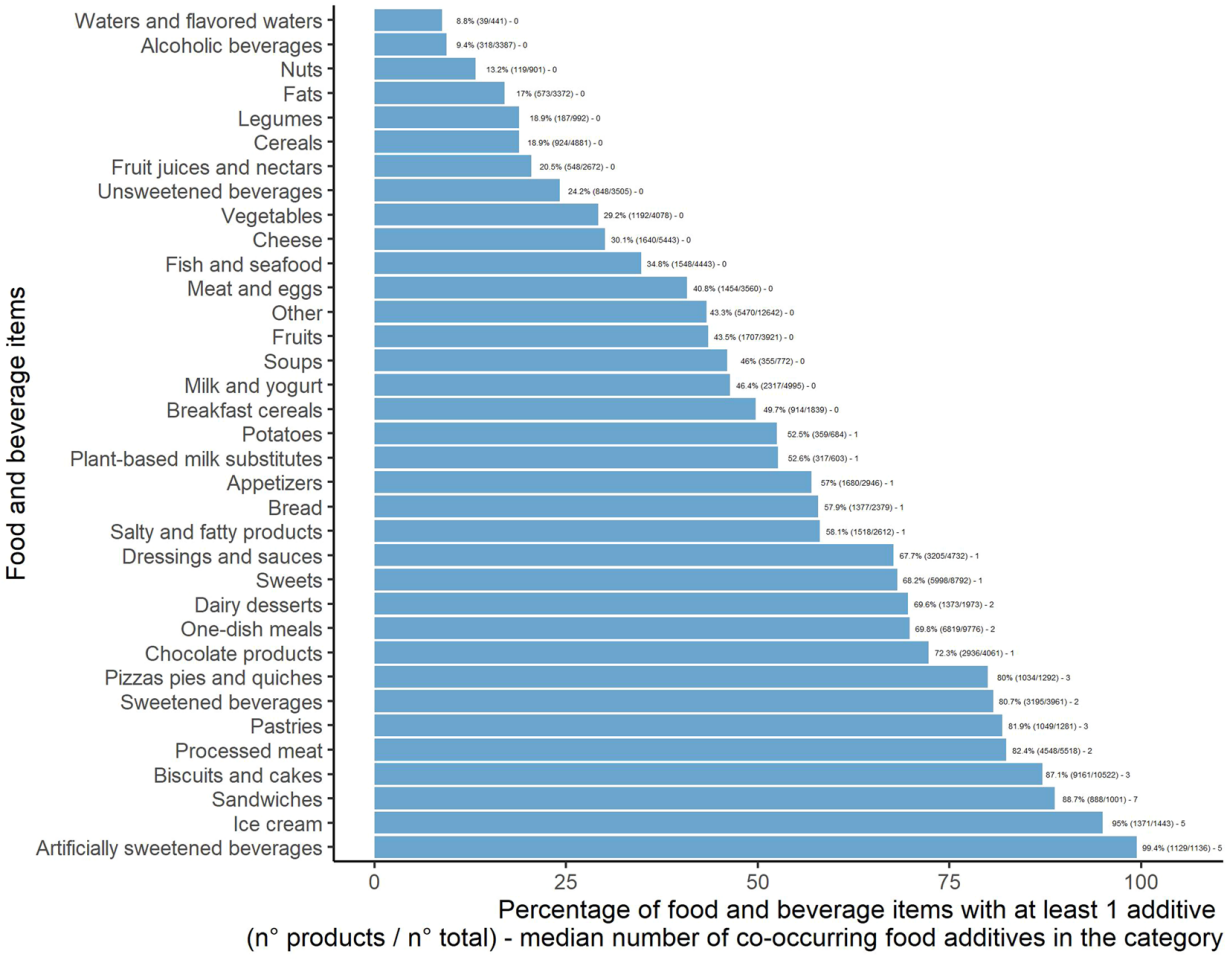
Percentage of food and beverage items containing at least one additive per food category, Open Food Facts database (n = 126,556 products), France 2019.

Figure 3 shows the percentage of products containing food additives, per food category. Virtually all artificially sweetened beverages (99.4% of products), 95.0% of ice creams, 88.7% of industrial sandwiches, and 87.1% of biscuits and cakes contained at least one food additive.

### Most frequently used food additives

**Figure 4:**
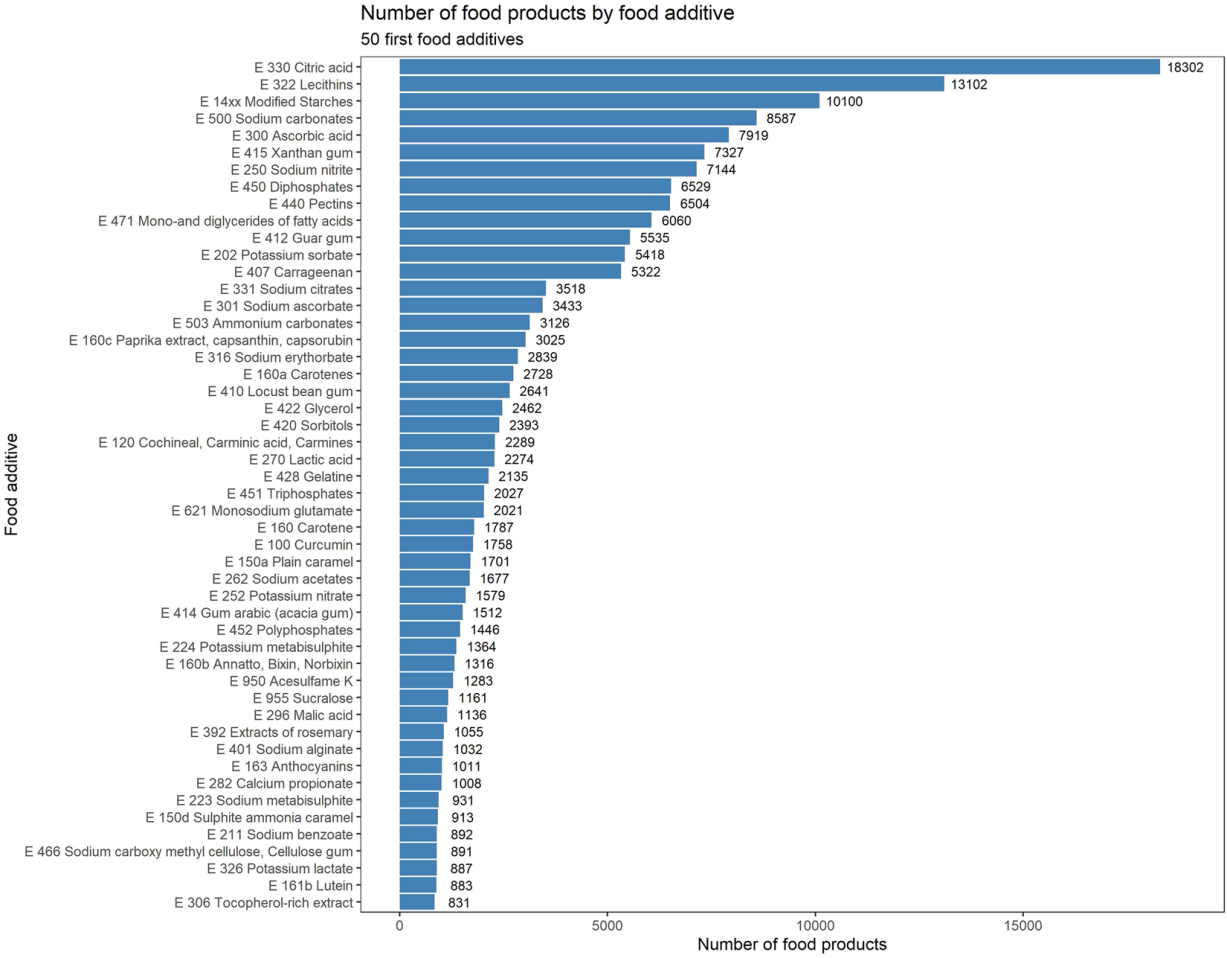
Number of food products containing each food additive, Open Food Facts database (n = 126,556 products), France 2019.

The number of food products containing each food additive is presented in Fig. 4 for the 50 most spread additives and in Appendix 7 for all food additives authorized on the EU market. The most frequently used food additives were citric acid, lecithins and modified starches (found in >10,000 products). The top 50 also included sodium nitrite, potassium nitrate, carrageenan, monosodium glutamate, sulfite ammonia caramel, acesulfame K, sucralose, (di/tri/poly)phosphates, mono- and diglycerides of fatty acids, potassium sorbate, cochineal, potassium metabisulphite, sodium alginate, bixin and sodium carboxymethylcellulose.

### Clusters of food additives frequently co-occurring in food products

**Figure 5:**
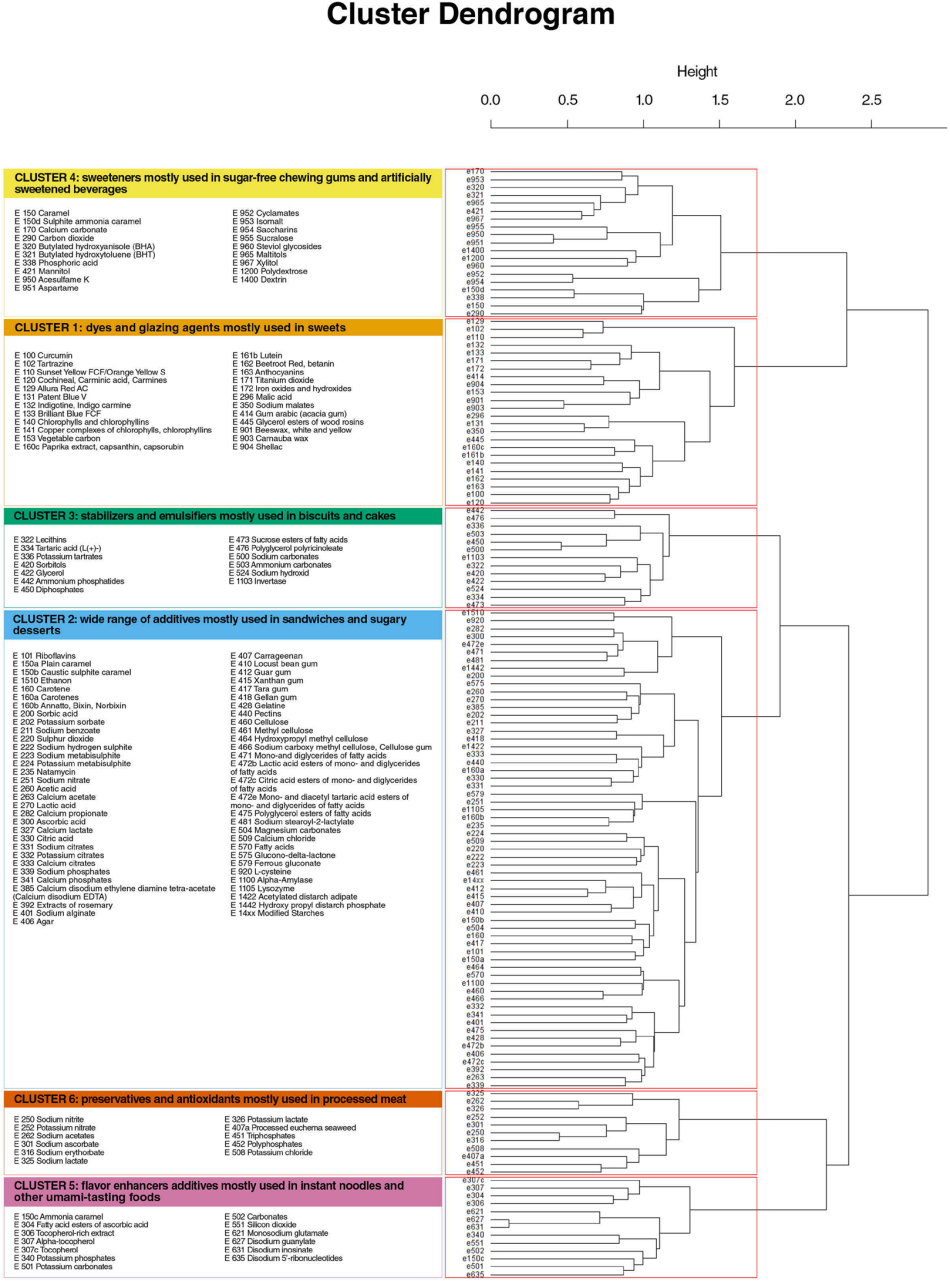
Dendrogram of food additives frequently co-occurring in food products generated by cluster analysis, Open Food Facts database, France 2019.

**Figure 6:**
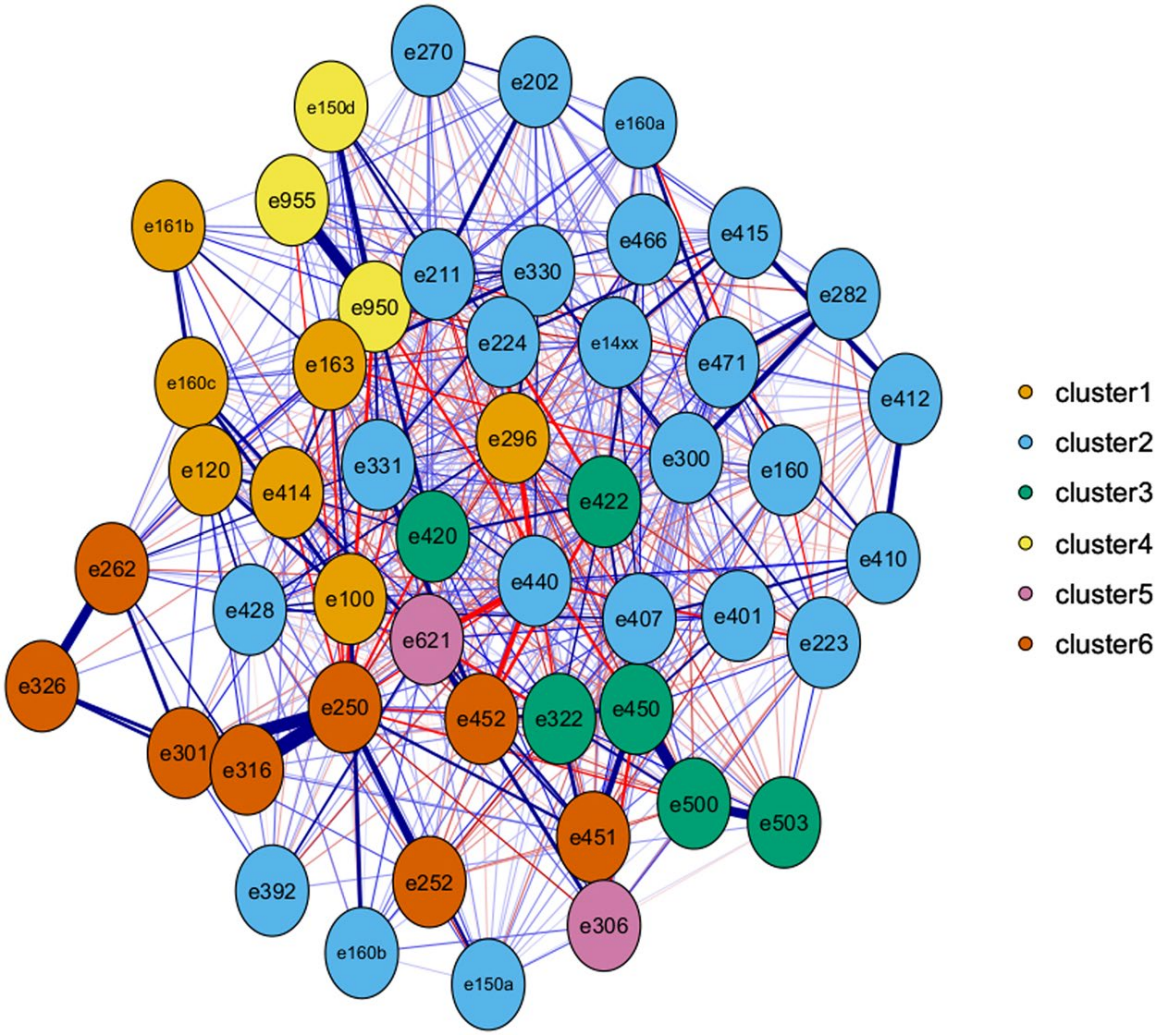
Network of food additives frequently co-occurring in food products generated by eLasso method, Open Food Facts database, France 2019.

After graphic assessment (Appendix 8a), 6 clusters of food additives were extracted (Fig. 5). Appendix 8b shows food additives present in each cluster and their respective squared loadings. Table 1 shows the 50 products most characteristic of each cluster, i.e., with the highest cluster synthetic variable (score). Network analysis also illustrated the co-occurrence of food additives (Fig. 6) and confirmed the clusters described here. The clusters were described as follows:

**Table 1:** Foods and beverage items most representative of each cluster of food additives, Open Food Facts database, France 2019*.

Cluster 1: dyes and glazing agents mostly used in sweets					Cluster 2: wide range of additives mostly used in sandwiches and sugary desserts					Cluster 3: stabilizers and emulsifiers mostly used in biscuits and cakes				
Product n°	Food group	Generic name	Cluster score	Nutri-Score	Product n°	Food group	Generic name	Cluster score	Nutri-Score	Product n°	Food group	Generic name	Cluster score	Nutri-Score
1	Chocolate products	chocolate	49,03	E	1	Biscuits and cakes	pastry	21,67	D	1	Biscuits and cakes	éclair	14,07	D
2	Chocolate products	chocolate	49,03	E	2	Biscuits and cakes	pastry	21,17	D	2	Biscuits and cakes	cake	13,56	E
3	Chocolate products	chocolate	45,31	E	3	Sandwiches	fish burger	18,22	D	3	Biscuits and cakes	chocolate cake	13,56	E
4	Sweets	chocolate	43,53	E	4	Sandwiches	burger	18,08	D	4	Biscuits and cakes	chocolate cake	13,56	E
5	Chocolate products	chocolate	40,17	E	5	Ice cream	vanilla ice cream	17,47	D	5	Biscuits and cakes	strawberry cake	12,89	D
6	Sweets	candy	39,89	D	6	Ice cream	chocolate ice cream	17,47	D	6	Biscuits and cakes	chocolate cake	12,89	D
7	Sweets	candy	39,89	D	7	Sandwiches	burger	16,90	D	7	Biscuits and cakes	chocolate cake	12,89	D
8	Chocolate products	chocolate	39,72	E	8	Sandwiches	burger	16,90	D	8	Biscuits and cakes	chocolate cake	12,89	D
9	Sweets	candy	39,24	D	9	Biscuits and cakes	pastry	16,71	D	9	Biscuits and cakes	éclair	12,89	D
10	Sweets	candy	39,24	D	10	Biscuits and cakes	pastry	16,55	D	10	Biscuits and cakes	chocolate cake	12,89	D
11	Sweets	candy	37,06	D	11	Biscuits and cakes	pastry	16,53	C	11	Biscuits and cakes	pastry	12,86	E
12	Sweets	candy mix	35,41	D	12	Biscuits and cakes	pastry	16,31	E	12	Biscuits and cakes	biscuits	12,38	E
13	Chocolate products	chocolate	35,41	C	13	Sandwiches	burger	16,02	D	13	Biscuits and cakes	biscuits	12,38	E
14	Chocolate products	chocolate	35,35	E	14	Biscuits and cakes	pastry	15,44	C	14	Biscuits and cakes	pastry	12,01	D
15	Chocolate products	chocolate	35,35	E	15	Sandwiches	burger	15,40	D	15	Biscuits and cakes	pastry	12,01	E
16	Sweets	candy mix	35,31	D	16	Pastries	brioche	15,32	D	16	Biscuits and cakes	pastry	12,01	D
17	Sweets	candy mix	34,89	D	17	Pastries	brioche	15,32	D	17	Biscuits and cakes	chocolate cake	11,90	E
18	Sweets	candy mix	34,89	D	18	Ice cream	ice cream	15,12	C	18	Biscuits and cakes	chocolate cake	11,90	E
19	Sweets	candy mix	34,82	D	19	Sandwiches	burger	15,11	D	19	Biscuits and cakes	biscuits	11,90	E
20	Sweets	candy mix	34,68	D	20	Sandwiches	burger	14,92	D	20	Biscuits and cakes	chocolate cake	11,90	E
21	Sweets	candy mix	34,68	D	21	Sandwiches	burger	14,92	D	21	Biscuits and cakes	chocolate cake	11,90	E
22	Sweets	candy mix	34,68	D	22	Biscuits and cakes	pastry	14,91	D	22	Biscuits and cakes	assorted biscuits	11,82	E
23	Sweets	candy mix	34,68	D	23	Biscuits and cakes	macaroon	14,89	D	23	Biscuits and cakes	biscuits	11,82	E
24	Sweets	candy mix	34,68	D	24	Dairy desserts	pastry	14,70	C	24	Biscuits and cakes	biscuits	11,82	E
25	Sweets	candy mix	34,68	D	25	Dairy desserts	pastry	14,70	C	25	Biscuits and cakes	biscuits	11,75	E
26	Sweets	candy mix	34,68	D	26	Biscuits and cakes	pastry	14,69	D	26	Biscuits and cakes	strawberry cake	11,70	D
27	Sweets	candy mix	34,68	D	27	Sandwiches	sandwich	14,68	D	27	Biscuits and cakes	Japaneese cake	11,70	D
28	Ice cream	ice cream	34,37	D	28	Biscuits and cakes	raspberry pastry	14,39	D	28	Biscuits and cakes	pastry	11,69	D
29	Ice cream	vanilla ice cream	34,06	C	29	Biscuits and cakes	brioche	14,21	D	29	Biscuits and cakes	pastry	11,59	D
30	Sweets	candy	33,69	D	30	Biscuits and cakes	pastry	14,20	C	30	Biscuits and cakes	pastry	11,59	D
31	Sweets	candy mix	33,23	D	31	Dairy desserts	coco mousse	14,15	C	31	Biscuits and cakes	éclair	11,59	D
32	Sweets	candy	32,95	D	32	Sandwiches	burger	14,12	D	32	Biscuits and cakes	éclair	11,59	D
33	Sweets	candy	32,95	D	33	Biscuits and cakes	strawberry pastry	14,10	C	33	Biscuits and cakes	pastry	11,44	D
34	Sweets	candy	32,95	D	34	Biscuits and cakes	pastry	14,09	D	34	Biscuits and cakes	chocolate cake	11,32	D
35	Sweets	candy	32,82	E	35	Ice cream	baked Alaska	13,99	D	35	Biscuits and cakes	chocolate cake	11,32	D
36	Sweets	candy	32,32	E	36	Ice cream	fruit ice cream	13,92	D	36	Biscuits and cakes	chocolate cake	11,32	D
37	Chocolate products	chocolate	32,10	E	37	Sandwiches	fish burger	13,86	D	37	Biscuits and cakes	éclair	11,21	D
38	Sweets	candy	31,92	D	38	Ice cream	fruit ice cream	13,78	C	38	Biscuits and cakes	éclair	11,21	D
39	Sweets	candy mix	31,64	D	39	Dairy desserts	lemon entremets	13,72	E	39	Biscuits and cakes	chocolate cake	11,21	D
40	Sweets	candy	31,23	D	40	Biscuits and cakes	pastry	13,60	D	40	Biscuits and cakes	chocolate cake	11,21	D
41	Chocolate products	chocolate	31,22	D	41	Other	pastry	13,56	D	41	Biscuits and cakes	chocolate cake	11,18	E
42	Chocolate products	chocolate	30,81	E	42	Biscuits and cakes	pastry	13,52	D	42	Biscuits and cakes	biscuits	11,09	C
43	Sweets	candy mix	30,28	C	43	Sandwiches	burger	13,51	D	43	Biscuits and cakes	chocolate cake	11,06	E
44	Sweets	candy	29,78	E	44	Sandwiches	sandwich	13,44	D	44	Biscuits and cakes	pastry	10,82	C
45	Sweets	candy mix	29,56	D	45	Biscuits and cakes	pastry	13,40	D	45	Biscuits and cakes	pastry	10,82	D
46	Sweets	candy	28,96	E	46	Sandwiches	sandwich	13,36	D	46	Biscuits and cakes	pastry	10,82	C
47	Chocolate products	chocolate	28,96	E	47	Sandwiches	chicken sandwich	13,36	D	47	Biscuits and cakes	pastry	10,82	D
48	Biscuits and cakes	biscuits	28,60	D	48	Fish and seafood	fish salad	13,35	C	48	Biscuits and cakes	pastry	10,69	E
49	Sweets	candy	28,54	D	49	One-dish meals	crepe	13,28	D	49	Biscuits and cakes	pastry	10,69	E
50	Sweets	candy	28,29	D	50	Pastries	brioche	13,23	D	50	Biscuits and cakes	pastry	10,69	E
**Cluster 4: sweeteners mostly used in sugar-free chewing gums and artificially sweetened beverages**					**Cluster 5: flavor enhancers additives mostly used in instant noodles and other umami-tasting foods**					**Cluster 6: preservatives and antioxidants mostly used in processed meat**				
**Product n°**	**Food group**	**Generic name**	**Cluster score**	**Nutri-Score**	**Product n°**	**Food group**	**Generic name**	**Cluster score**	**Nutri-Score**	**Product n°**	**Food group**	**Generic name**	**Cluster score**	**Nutri-Score**
1	Sweets	chewing gum with sweeteners	49,23	B	1	One-dish meals	instant noodles	35,75	E	1	One-dish meals	pork macaroni gratin	19,95	C
2	Sweets	chewing gum with sweeteners	49,23	B	2	One-dish meals	instant noodles	33,48	C	2	One-dish meals	endive gratin with pork	19,95	C
3	Sweets	chewing gum with sweeteners	48,53	D	3	One-dish meals	instant noodles	33,27	C	3	Processed meat	diced ham	19,48	D
4	Sweets	chewing gum with sweeteners	48,53	B	4	One-dish meals	instant noodles	32,76	C	4	Sandwiches	croque-monsieur	19,38	D
5	Sweets	chewing gum with sweeteners	48,53	B	5	One-dish meals	instant noodles	32,53	C	5	Pizzas pies and quiches	quiche with ham	18,61	D
6	Sweets	chewing gum with sweeteners	46,59	B	6	One-dish meals	instant noodles	32,53	C	6	Pizzas pies and quiches	quiche with ham	18,61	D
7	Sweets	chewing gum with sweeteners	46,59	B	7	One-dish meals	instant noodles	31,83	E	7	Pizzas pies and quiches	quiche with ham	18,61	D
8	Sweets	chewing gum with sweeteners	46,59	B	8	One-dish meals	instant noodles	31,41	C	8	Processed meat	diced ham	18,18	D
9	Sweets	chewing gum with sweeteners	46,59	B	9	One-dish meals	instant noodles	31,32	C	9	Pizzas pies and quiches	quiche with ham	17,76	D
10	Sweets	chewing gum with sweeteners	46,59	B	10	One-dish meals	instant noodles	31,32	C	10	One-dish meals	sauerkraut	17,55	D
11	Sweets	chewing gum with sweeteners	46,59	B	11	One-dish meals	instant noodles	31,20	C	11	One-dish meals	cassoulet	17,55	B
12	Sweets	chewing gum with sweeteners	46,59	B	12	One-dish meals	instant noodles	31,20	C	12	Processed meat	sauerkraut	17,55	D
13	Sweets	chewing gum with sweeteners	46,59	B	13	One-dish meals	instant noodles	30,69	C	13	One-dish meals	Cantonese rice with ham	17,02	D
14	Sweets	chewing gum with sweeteners	46,59	D	14	One-dish meals	instant noodles	30,69	C	14	Pizzas pies and quiches	quiche with ham	16,24	B
15	Sweets	chewing gum with sweeteners	46,59	B	15	One-dish meals	instant noodles	29,28	C	15	Pizzas pies and quiches	quiche with ham	15,96	D
16	Sweets	chewing gum with sweeteners	46,59	B	16	One-dish meals	instant noodles	29,25	C	16	Pizzas pies and quiches	quiche with ham	15,96	D
17	Other	chewing gum with sweeteners	46,59	C	17	One-dish meals	instant noodles	29,25	C	17	Pizzas pies and quiches	quiche with ham	15,96	C
18	Sweets	chewing gum with sweeteners	46,59	C	18	One-dish meals	instant noodles	29,25	C	18	Pizzas pies and quiches	quiche with ham	15,96	D
19	Sweets	chewing gum with sweeteners	46,59	C	19	One-dish meals	instant noodles	28,82	C	19	One-dish meals	crepe with ham	15,83	D
20	Sweets	chewing gum with sweeteners	46,59	B	20	One-dish meals	instant noodles	28,82	C	20	Processed meat	chicken ham	15,81	C
21	Sweets	chewing gum with sweeteners	46,59	B	21	One-dish meals	instant noodles	28,82	C	21	Meat and eggs	turkey ham	15,81	C
22	Sweets	chewing gum with sweeteners	46,59	B	22	One-dish meals	instant noodles	28,08	C	22	Processed meat	chicken ham	15,81	C
23	Sweets	chewing gum with sweeteners	46,59	B	23	Other	dried broth	27,61	D	23	Meat and eggs	pork roulades	15,61	D
24	Sweets	chewing gum with sweeteners	46,59	B	24	One-dish meals	instant noodles	27,49	C	24	Processed meat	sausage	15,61	D
25	Sweets	chewing gum with sweeteners	46,59	B	25	One-dish meals	instant noodles	27,40	C	25	Processed meat	sausage	15,61	E
26	Sweets	chewing gum with sweeteners	46,59	C	26	One-dish meals	instant noodles	27,40	C	26	Processed meat	sausage	15,61	E
27	Sweets	chewing gum with sweeteners	46,47	B	27	One-dish meals	instant noodles	27,40	C	27	Processed meat	sausage	15,61	E
28	Sweets	chewing gum with sweeteners	46,47	B	28	One-dish meals	instant noodles	27,40	C	28	Processed meat	sausage	15,61	E
29	Sweets	chewing gum with sweeteners	45,78	B	29	One-dish meals	instant noodles	26,98	C	29	Processed meat	sausage	15,61	E
30	Sweets	chewing gum with sweeteners	44,54	B	30	One-dish meals	instant noodles	26,98	E	30	Processed meat	sausage	15,61	D
31	Sweets	chewing gum with sweeteners	43,79	B	31	One-dish meals	instant noodles	26,98	C	31	Processed meat	sausage	15,61	E
32	Sweets	chewing gum with sweeteners	43,79	B	32	One-dish meals	instant noodles	26,98	C	32	Processed meat	sausage	15,61	D
33	Sweets	chewing gum with sweeteners	43,79	B	33	One-dish meals	instant noodles	26,98	C	33	Processed meat	sausage	15,61	D
34	Sweets	chewing gum with sweeteners	43,79	B	34	One-dish meals	instant noodles	26,98	C	34	Processed meat	sausage	15,61	E
35	Sweets	chewing gum with sweeteners	43,09	B	35	One-dish meals	instant noodles	26,98	C	35	Processed meat	sausage	15,48	E
36	Sweets	chewing gum with sweeteners	42,94	C	36	One-dish meals	instant noodles	26,98	C	36	One-dish meals	delicatessen product	15,48	D
37	Sweets	chewing gum with sweeteners	41,20	B	37	One-dish meals	instant noodles	26,98	C	37	Meat and eggs	cooked chicken slices	15,22	E
38	Sweets	chewing gum with sweeteners	41,20	B	38	One-dish meals	instant noodles	26,98	C	38	Processed meat	sausage	15,18	B
39	Sweets	chewing gum with sweeteners	41,20	C	39	One-dish meals	instant noodles	26,98	C	39	Processed meat	sausage	15,18	D
40	Sweets	chewing gum with sweeteners	40,30	B	40	One-dish meals	instant noodles	26,98	C	40	Processed meat	turkey ham	14,87	D
41	Sweets	chewing gum with sweeteners	39,81	B	41	One-dish meals	instant noodles	26,98	C	41	One-dish meals	tandoori chicken	14,87	D
42	Sweets	chewing gum with sweeteners	39,64	B	42	One-dish meals	instant noodles	26,98	C	42	Sandwiches	croque-monsieur	14,87	C
43	Sweets	chewing gum with sweeteners	39,64	B	43	One-dish meals	instant noodles	26,98	C	43	Cereals	ready to eat pastas with ham	14,78	D
44	Sweets	chewing gum with sweeteners	39,64	B	44	One-dish meals	instant noodles	26,98	D	44	Processed meat	delicatessen product	14,77	C
45	Sweets	chewing gum with sweeteners	39,60	B	45	One-dish meals	instant noodles	26,98	C	45	Processed meat	delicatessen product	14,77	D
46	Sweets	chewing gum with sweeteners	39,60	B	46	One-dish meals	instant noodles	26,78	C	46	Meat and eggs	rabbit roulades	14,62	D
47	Sweets	chewing gum with sweeteners	39,48	B	47	One-dish meals	instant noodles	26,78	C	47	Meat and eggs	rabbit roulades	14,62	D
48	Sweets	chewing gum with sweeteners	39,48	B	48	One-dish meals	instant noodles	26,78	C	48	One-dish meals	delicatessen product	14,44	D
49	Sweets	chewing gum with sweeteners	39,48	B	49	One-dish meals	instant noodles	26,78	C	49	One-dish meals	ham cake	14,24	D
50	Sweets	chewing gum with sweeteners	39,48	B	50	One-dish meals	instant noodles	26,75	C	50	Processed meat	turkey ham	14,24	D

*This table displays the 50 food or beverage items with the highest score (synthetic cluster variable) for each of the 6 food additive clusters. Details on food additive clusters are available in Appendix 8.

All food products in this table were categorized as NOVA “4”.

### Cluster 1: dyes and glazing agents mostly used in sweets (n = 24 food additives)

This cluster was mostly represented by carnauba wax (used to produce a glossy finish), beeswax (glazing agent), titanium dioxide (dye), curcumin (dye), brilliant blue FCF and patent blue V (dye). Foods most representative of this cluster (99^th^ percentile of the score for cluster 1) were sweets. The distribution of their Nutri-Score was 0.6% A, 14.0% B, 6.0% C, 56.2% D, 23.2% E and their repartition across NOVA categories was 0.1% 1 and 99.9% 4.

### Cluster 2: wide range of additives mostly used in sandwiches and sugary desserts (n = 61 food additives)

This cluster was mostly represented by xanthan gum, modified starches, mono- and diglycerides of fatty acids, guar gum and carrageenan. They have a wide range of functions, such as emulsifiers, stabilizers, colorings, antioxidants and are used in a wide variety of products. Foods most representative of this cluster (99^th^ percentile) were sandwiches and sugary desserts. The distribution of their Nutri-Score was 3.6% A, 9.2% B, 24.7% C, 49.9% D, 12.6% E and their repartition across NOVA categories was 0.1% 3, and 99.9% 4.

### Cluster 3: stabilizers and emulsifiers mostly used in biscuits and cakes (n = 13 food additives)

This cluster is mostly represented by sodium carbonate, diphosphates, lecithins, ammonium carbonates, and glycerol. These additives are mostly used as acidity regulators, stabilizers and emulsifiers. Foods most representative of this cluster (99^th^ percentile) were biscuits and cakes. The distribution of their Nutri-Score was 0.2% A, 3.5% B, 5.4% C, 46.0% D, 44.9% E and their repartition across NOVA categories was 100% 4.

### Cluster 4: sweeteners mostly used in sugar-free chewing gums and artificially sweetened beverages (n = 19 food additives)

This cluster is mostly represented by sweeteners such as acesulfame K, aspartame, xylitol, maltitols, mannitol and the antioxidant butylated hydroxytoluene (BHT). Foods most representative of this cluster (99^th^ percentile) were sweets (mostly chewing-gums with sweeteners) and artificially sweetened beverages. The distribution of their Nutri-Score was 13.1% A, 43.6% B, 20.3% C, 17.2% D, 5.8% E and their repartition across NOVA categories was 0.1% 2, 0.6% 3, and 99.3% 4.

### Cluster 5: flavor enhancers additives mostly used in instant noodles and other umami-tasting foods (n = 13 food additives)

This cluster is represented by flavor enhancers disodium inosinate, disodium guanylate and monosodium glutamate, used in synergy to provide the umami taste and potassium carbonates, disodium 5′-ribonucleotides and ammonia caramel. Foods most representative of this cluster (99^th^ percentile) were one-dish meals (mostly instant noodles) and appetizers (mostly salted crisps). The distribution of their Nutri-Score was 4.3% A, 10.8% B, 21.5% C, 42.1% D, 21.3% E and their repartition across NOVA categories was 1.3% 1, 0.2% 2, 1.9% 3 and 96.6% 4.

### Cluster 6: preservatives and antioxidants mostly used in processed meat (n = 11 food additives)

This cluster is mostly represented by sodium nitrite, sodium erythorbate, sodium ascorbate, triphosphates and sodium acetates. Sodium nitrite is a preservative used in a wide variety of processed meat. It can be used with sodium erythorbate, which increases the rate at which nitrite reduces to nitric oxide. Foods most representative of this cluster (99^th^ percentile) were processed meat and one-dish meals containing processed meat. The distribution of their Nutri-Score was 2.8% A, 9.1% B, 20.2% C, 42.5% D, 25.4% E and their repartition across NOVA categories was 0.8% 3, and 99.2% 4.

## Discussion

This paper provides for the first time an overview of the presence and co-occurrences of food additives in 126,556 packaged food products available on the French food market. Food additives were present in 53.8% of products and covered a wide variety of categories illustrating their widespread use in French manufactured products. More than 10% of products contained 5 or more food additives. We identified 6 clusters of food additives frequently co-occurring in food products that were confirmed by network analysis and which corresponded to additives typically found in sweets, sandwiches & sugary desserts, biscuits & cakes, sugar-free chewing-gums & artificially sweetened beverages, instant noodles & other umami-tasting foods, and processed meat. The most frequently used food additives were citric acid, lecithins and modified starches (>10,000 products). Other additives of the top 50 were: sodium nitrite, potassium nitrate, carrageenan, monosodium glutamate, sulfite ammonia caramel, acesulfame K, sucralose, (di/tri/poly)phosphates, mono- and diglycerides of fatty acids, potassium sorbate, cochineal, potassium metabisulphite, sodium alginate, bixin and sodium carboxymethylcellulose.

A recent study by the French food observatory “*Observatoire de l’Alimentation*” (Oqali) evaluated the frequency of use of certain food additives and the evolution between 2012 and 2018 in a selection of food products^61^. The most frequently used additives where consistent with our study (e.g. citric acid, modified starches and lecithins in the top 3; acesulfame K as the most used sweetener). Their report suggests a diminution of the use of food additives since 2012 in half of the food categories studied, but an increase in some food additives such as carotenoids used as colors but also some artificial sweeteners (sorbitol syrup, sucralose in sweet beverages), and sulfites in aperitive products and salsas. Our study was based on a larger sample of manufactured products present on the French market (126,556 vs 30,125) and covered all food groups, whereas some categories such as sweets, chewing gums, and confectionary were not analyzed in the Oqali report.

For several frequently used additives (i.e. pertaining to the “top 50” in the present study), potential adverse health effects have been suggested in *in-vivo/in-vitro*, and - more rarely - epidemiological studies. For instance, sodium nitrite and potassium nitrate (e250/e252, 7144/1579 food products, respectively) have been associated in prospective cohorts with all-cause mortality (nitrates/nitrites from preserved/processed meat)^23^, and gastric and pancreatic cancers^21,22^. Phosphates have been associated with vascular effects (e.g. endothelial dysfunction and vascular calcification) in experimental studies among humans^35,36^. Monosodium glutamate (e621, 2021 products) might have patho-physiological and toxicological effects on human health^25,27^ and was associated with overweight in a prospective cohort^26^. Sulfites (among which potassium metabisulphite, e224, 1364 products) have been associated with alteration of the gut and mouth microbiome *in vitro* at concentrations considered as safe for food^62^. Nonnutritive sweeteners such as acesulfame K, sucralose and aspartame (e950/e955/e951, 1283/1161/669 products, respectively) still have controversial effects on human cardiometabolic health and adiposity^30^ and have been linked with hematopoietic neoplasia and gut microbiota alteration in experimental studies on rodents^31–34^. Sulfite ammonia caramel (e150d, 913 products), present in almost every cola, might carry 4-methylimidazole (4-MEI) defined as possibly carcinogenic to humans by the International Agency for Research on Cancer, IARC^40,41^. Carrageenan (e407, 5322 products) has been linked to fasting hyperglycemia and with exacerbated glucose intolerance and hyperlipidemia without effect on weight among mice^24^. Carboxymethylcellulose (e466, 891 products) has been associated with changes in microbiota composition, intestinal inflammation and metabolic syndrome (*in-vivo*)^37,63–65^, pro-inflammation (*in-vivo*, *ex-vivo*)^66–69^ and promotion of tumor development (*in-vivo*)^38^. Finally, an experimental study among humans suggests a link between lecithins (e322, 13102 products) and coronary artery disease through the production of a proatherosclerotic metabolite, trimethylamine-N-oxide (TMAO)^70^. On the other hand, some food additives could be candidates for beneficial long-term health effects. For instance, bixin (e160b, 1316 products) has shown reduction of postprandial inflammatory and oxidative stress responses to high-calorie meals in a human randomized-controlled trial^71^. Furthermore, ascorbic acid (e300, 7919 products) used as an antioxidant might beneficially contribute to total ascorbic acid intake, as suggested by EFSA combined exposure assessment^72^). Also, some food additives such as extracts of rosemary (e392, 1055 products) could also be of interest as many of their components are phenolic acids. Finally, sodium alginate (e401, 1032 products) has been suggested to improve liver steatosis, insulin resistance, chronic inflammation, and oxidative stress, preventing the development of liver tumorigenesis among obese and diabetic mice^73^. Contrasted health effects have been suggested in experimental data for modified starches. For instance, distarch phosphate (e1412) and hydroxypropyl-distarch phosphate (e1442) showed potential beneficial effects on postprandial glycaemia and insulin response in human trials^74,75^. In contrast, maize hydroxypropylated distarch phosphate (e1442) promoted a less varied microbiota in faeces of healthy infant donors of 2–3 months of age^76^.

The two methods used to identify food additives frequently co-occurring in food products were complementary. In the IsingFit methods, the edges in a network represent conditional dependencies: if there is an edge between additive X1 and X2, they are related even after controlling for all other connections in the network. If two variables X2 and X3 are not connected in the network representation, it means that they are not directly related, but they might be correlated by sharing connections with other variables in the network. When conditioned on all other variables, the relationship between X2 and X3 disappears — it is explained away by the other variables. On the other side, the ClustOfVar method groups together variables which are strongly related to each other (directly or indirectly) and does not adjust for other variables as this is not the aim of a clustering method. Despite small discrepancies due to these methodological differences, the two methods provided overall consistent results, as highlighted when the network obtained by *eLasso* was colored according to the clusters generated by the ClustOfVar method (Fig. 6).

Each food additive cluster occurred in several broad food sectors. Among the 6 clusters of food additives frequently co-occurring in food products identified in the present study, 2 clusters were found mostly in salty products (instant noodles and processed meat) and 4 clusters occurred mainly in sweet products (sweets, sandwiches & sugary desserts, sugar-free chewing-gums & artificially sweetened beverages, and biscuits & cakes). The additives that constituted each cluster had sometimes complementary functional properties. For instance in cluster 3, main food additives where sodium carbonate (e500), diphosphates (e450), ammonium carbonates (e503) and glycerol (e422) which are widely used in biscuits and cakes as acidity regulators, stabilizers and emulsifiers.

More than 10% of manufactured products contained 5 or more additives. Thus, consumers are regularly exposed to mixtures of food additives, but this multiple exposure has rarely been studied in the literature. For instance, the 3 first food additives of cluster 3 (sodium carbonate (e500), diphosphates (e450) and lecithins (e322)) co-occur in 1667 products, mostly biscuits. Also, the cumulative exposure to the 3 first food additives of cluster 4 (acesulfame K (e950), aspartame (e951) and xylitol (e967)) could be of interest as these three additives co-occur in 87 products in our study sample, among which some very popular sugar-free chewing-gums.

So far, detailed information on potential cocktail effects is lacking but several studies started to suggest interactions and synergies between food additives. For example, mixes of colorings with sodium benzoate (e211) were associated with increased hyperactivity in 3-year-old and 8/9-year-old children^45^. Neurotoxic effects were also observed between combinations of brilliant blue (e133) with L-glutamic acid (e620), and quinoline yellow (e104) with aspartame (e951) *in vitro*^46^ and a mixture of food coloring additives increased oxidative stress in rats^47^. Future prospective studies and experimental research should investigate the effects of chronic exposures to these cocktails of food additives, as consumed in real life.

Strengths of our study included the large number of food and beverage items displayed by the Open Food Facts database and the originality of the cluster and network-based statistical approach applied to depict the food additives frequently co-occurring in food products. Conversely, some limitations should be acknowledged. First, the collaborative Open Food Facts database does not exhaustively cover all industrial food items available on the French market. Such exhaustive formulation database does not exist so far. However, it provides an extensive coverage with nearly 130,000 complete references of different products, and its crowdsourced nature by consumers guarantees the fact that all frequently consumed products are registered. Second, as any contributor-based data, some errors in food composition may not be excluded and some formulation data may have evolved since their entry in the database. However, despite the fact that the information is contributor-based, errors are minimized by performant text and picture recognition algorithms allowing automated checks. Besides, systematic control campaigns including random sampling and checking of food products as well as update of information (taking into account reformulations) are regularly performed. Moreover, an increasing number of manufacturers regularly implement their validated data directly in the system. Third, this study focused on the presence/absence of additives in food products, without data on the doses of additives used. Such information is not mandatory on food labels in the current regulation. Using the relative position of additives in the ingredient list would provide very little information on the real additive amounts. Indeed, additives are generally all mentioned at the end of the ingredient list since their amounts are much lower than main ingredients (flour, sugar, etc.). Regarding relative amounts between additives, ingredient list would bring no information for all food products with only one additive (i.e. 33% of products containing additives). Besides, even if the order of additives in the ingredients’ list is similar between two food products, it may represent very different absolute amounts of additives in the two products. However, the paradigm “*the dose makes the poison*” is currently being challenged in toxicology, with effects at very low doses suggested for many chemical substances. Thus, the information on presence/absence of additives is already interesting in itself. Next, the Open Food Facts database does not provide information on sales, so it was not possible to weight the analysis according to purchase volumes or frequency. To obtain a complete picture of mixtures of food additives frequently ingested, the next step will be to merge the present composition data with detailed consumption data (which is even more accurate than sales data at the individual level). Finally, on food packaging, the detail on the type of modified starch is often not specified and only the broad category “modified starch” is mentioned. Thus, the number of products containing each of the specific modified starches may be underestimated in the database.

This study illustrates the widespread of food additives in foods available on the French market and allowed us to identify 6 clusters of frequently co-occurring food additives. It is important that this type of industrial food composition database remains open-access and freely available to guarantee the transparency for the consumers and for public researchers and stakeholders, making it possible to monitor the trends in food additive use and to perform etiological research. To concretely expand etiological research on food additives and health, doses of additives should also be made available in complete transparency. As a key perspective of this study, our research team is currently launching a large-scale program on chronic exposure to mixtures of food additives and health that will notably rely on the NutriNet-Santé cohort^77^, in which commercial names and brands of industrial products consumed are reported by the participants through detailed and repeated dietary records, which is crucial for an accurate evaluation of exposure at the individual level, due to the high variability in additive composition between brands for a similar type of product. Within this framework, we have planned different strategies to collect quantitative information on food additive content, including ad hoc laboratory assays of strategic additives in specific food matrixes and pro-active collection of dose data from manufacturers. In the meanwhile and following the precautionary principle, French health authorities recommend to limit the consumption of ultra-processed foods and, in practice, to choose food products with a better nutritional quality (as scored by the Nutri-Score) and with no or as few additives as possible.

## Supplementary information

Supplementary information.
