# Supplementary material for: Food additives: distribution and co-occurrence in 126,000 food products of the French market
Source: Sci Rep. 2020 Mar 4;10:3980. doi: 10.1038/s41598-020-60948-w (PMC7055242; doi:10.1038/s41598-020-60948-w)
Supplement: Supplementary file 1 — Supplementary information. [file 41598_2020_60948_MOESM1_ESM.pdf]

APPENDIX

Food additives: distribution and co-occurrence in 126 000 food products of the French market.

Eloi Chazelas[1], Mélanie Deschasaux[1], Bernard Srour[1], Emmanuelle Kesse-Guyot[1], Chantal Julia[1,2], Benjamin Alles[1], Nathalie Druesne-Pecollo[1], Pilar Galan[1], Serge Hercberg[1,2], Paule Latino-Martel[1], Younes Esseddik[1], Fabien Szabo[1], Pierre Slamich[3], Stephane Gigandet[3], Mathilde Touvier[1]*

Affiliations:

[1] Sorbonne Paris Nord - Paris 13 University, Inserm U1153, Inrae U1125, Cnam, Nutritional Epidemiology Research Team (EREN), Epidemiology and Statistics Research Center – University of Paris (CRESS), Bobigny, France

[2] Public Health Department, Avicenne Hospital, AP-HP, Bobigny, France

[3] Open Food Facts, France

***Corresponding author:**

Eloi Chazelas

Sorbonne Paris Nord - Paris 13 University, Inserm U1153, Inrae U1125, Cnam, Nutritional Epidemiology Research Team (EREN), Epidemiology and Statistics Research Center – University of Paris (CRESS)

SMBH Paris 13, 74 rue Marcel Cachin F-93017 Bobigny Cedex, France

E-mail: e.chazelas@eren.smbh.univ-paris13.fr

ORCID: 0000-0002-4156-0026

Tel: +33 1 48 38 73 78

Appendix 1: Flowchart

379 661 products from Open Food Facts World, April 2019

↓ Exclusion of 239 872 products

139 789 products marketed in France with provided list of ingredients

↓ Exclusion of 13 233 duplicates

126 556 products included

Appendix 2: Description of broad food categories, Open Food Facts database (n=126 556 products), France 2019

Food category	Number of products	Description
Alcoholic beverages	3387	All types of alcoholic beverages
Artificially sweetened beverages	1136	All types of artificially sweetened beverages, including protein mixes with sweeteners and syrups with sweeteners
Fruit juices and nectars	2672	All types of fruit juices and nectars, including 100% fruit juices
Plant-based milk substitutes	603	Soy/almonds/rice drinks, coconut milks, cereal-based drinks
Sweetened beverages	3961	Sodas, sweetened iced teas and coffees, syrups
Unsweetened beverages	3505	Vegetables drinks, coffee and tea
Waters and flavored waters	441	Unsweetened water, still or sparkling
Bread	2379	Bread, toasts, croutons
Breakfast cereals	1839	Sugary sweetened cereals, muesli, oat flakes, puffed cereals, cereal bars
Cereals	4881	Pasta, rice, flour
Legumes	992	Lentils, beans, chikpeas
Potatoes	684	Potatoes, sweet potatoe, plantain banana, igname
One-dish meals	9776	Ready-made meals, composite salads
Pizza pies and quiche	1292	Pizza, vegetable pie, meat pie, quiche
Sandwiches	1001	Sandwich, hamburger, panini
Dressings and sauces	4732	Salad dressing, bechamel sauce, mayonnaise, pesto sauce
Fats	3372	Butter, vegetable oils
Meat and eggs	3560	Beef, lamb, turkey, chicken, duck, rabbit, pork, eggs, kidneys, liver, tripes, beef tongue, blood pudding
Fish and seafood	4443	Salmon, tuna fish, cod, sole, t-burbot, skate, herring, sardines, anchovies, shrimps, scallops, oysters, mussels
Processed meat	5518	Ham, salami, pâté, bacon, sausages
Fruits	3921	Raw and cooked fruits, fruits in syrup, dried fruit
Soups	772	Soups, dry soups, minestrone, broth
Vegetables	4078	Raw and cooked vegetables
Cheese	5443	All types of cheese
Dairy desserts	1973	Batter pudding, mousse, custard, crème brulée, crème caramel, flan, pannacotta, rice puddings
Ice cream	1443	Ice cream, sorbet
Milk and yogurt	4995	Milk, cottage cheese, yogurt
Appetizers	2946	Potatoe chips, crackers
Nuts	901	Salted and unsalted nuts
Salty and fatty products	2612	Savory spreads such as houmous, tzatziki, tarama
Biscuits and cakes	10522	chocolate cakes, cookies, sugary pie, pancakes, clafoutis, biscuits, muffins, gingerbread, meringue, sponge cake, donuts
Chocolate products	4061	Chocolate, chocolate bars, chocolate spread
Pastries	1281	Croissants, chocolate croissants, brioche
Sweets	8792	Jam, honey, sweets, chewing gums
Other	12642	Products that could not be classified (dry tea, instant coffee, spices, aromatic herbs, etc.)

Appendix 3: Description of the ClustOfVar algorithm used for variable clustering

The ClustOfVar algorithm aims at maximizing a homogeneity criterion: clusters are defined as homogeneous when the clustered variables are strongly linked (measured here by correlation ratios) to a central quantitative synthetic variable[1]. The synthetic variable of a cluster is the first principal component of PCAMIX applied to all variables of the cluster. Thus, it is a linear combination of the variables of the cluster[2]. For a given food product, the value of this synthetic variable for $cluster_i$ is directly correlated to the number of food additives of $cluster_i$ present in this food.

As stated in Chavent and colleague's article[1], synthetic variable of a cluster C_k is defined as the quantitative variable c_k the "most linked" to all the variables in C_k:

$$\mathbf{c}_k = \arg\max_{\mathbf{u}\in\mathcal{R}^n} \left\{ \sum_{\mathbf{x}_j\in C_k} r^2_{\mathbf{u},\mathbf{x}_j} + \sum_{\mathbf{y}_j\in C_k} \eta^2_{\mathbf{u}|\mathbf{y}_j} \right\},$$

where r^2 denotes the squared Pearson correlation and η^2 denotes the correlation ratio. More precisely, the correlation ratio measures the part of the variance of u explained by the categories of y_i:

$$\eta^2_{\mathbf{u}|\mathbf{y}_j} = \frac{\sum_{s\in\mathcal{M}_j} n_s(\bar{\mathbf{u}}_s - \bar{\mathbf{u}})^2}{\sum_{i=1}^{n}(u_i - \bar{\mathbf{u}})^2},$$

where n_s is the frequency of category s, $\bar{\mathbf{u}}_s$ is the mean value of **u** calculated on the observations belonging to category s and $\bar{\mathbf{u}}$ is the mean of **u**.

For each cluster, a squared loading is attributed to each additive, corresponding to the correlation ratio between the food additive and the central synthetic variable of the cluster. A bootstrap process was performed to evaluate the stability of the partitions of variables and to determine a suitable number of clusters.

Appendix 4: Description of the R package IsingFit used for network analysis

The R package IsingFit[3] performs network analysis with an approach based on Ising models[4] that aims to estimate two parameters: interaction parameters which represents the strength of the interaction between two variables, and the node parameter, which represents the autonomous disposition of the variable to take the value one, regardless of neighboring variables. These parameters are estimated with logistic regressions, one variable being regressed on all other iteratively, and a ℓ_1 penalty (*lasso*) is applied to the regression parameters. The level of shrinkage depends on the penalty parameter of the lasso, which is determined by the *eLasso* method[3], based on the Extended Bayesian Information Criterion (EBIC)[5]. *eLasso* has proven to be an effective method to estimate networks from binary data, and simulations indicated that the probability of including an edge that is not present in the generating network is very small.

Appendix 5: Categorization of food items of the Open Food Facts database according to their nutritional quality scored by the Foods Standard Agency Nutrient Profilng system (FSAm- NPS)

The Nutri-Score was selected by the French, the Spanish and the Belgian Ministries of Health as the official front-of-pack nutrition label to be implemented in these countries[6]. It is based on a modified version of the British Food Standards Agency Nutrient Profiling System (FSAm-NPS) and allows to categorize food products into 5 colours reflecting their nutritional quality (from A- green: best nutritional quality to E-red lower nutritional quality). It takes into account the content per 100g of energy, saturated fatty acids, sugar, sodium, dietary fibres, proteins and fruit/vegetables[7]: The FSAm-NPS score was available in Open Food Facts for 107302/126556 (84.8%) products and was calculated as follows: points (0–10) are allocated for the content per 100 g in total sugars (g), saturated fatty acids (g), sodium (mg), and energy (kJ) (i.e., nutrients that should be consumed in limited amounts) and can be balanced by opposite points (0–5) allocated for dietary fibres (g), proteins (g), and fruits/vegetables/legumes/nuts (percent) (i.e., nutrients/components that should be promoted). The FSAm-NPS score for each food/beverage is based on a unique discrete continuous scale ranging theoretically from −15 (most healthy) to +40 (least healthy). The detailed algorithm has been published[8].

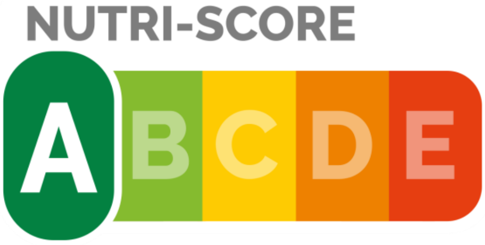

Distribution of food items according to Nutri-score categories, Open Food Facts database, France 2019

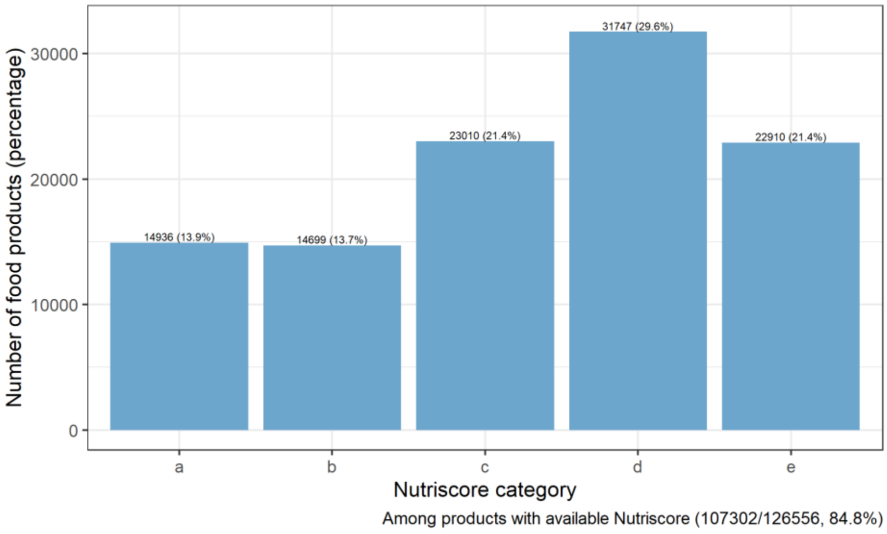

Appendix 6: Categorization of food items of the Open Food Facts database according to the NOVA classification

All food and beverage items of the Open Food Facts database were categorized into one of the four food groups in NOVA, a food classification system based on the extent and purpose of industrial food processing[9–11]. NOVA groups were as follows: "unprocessed or minimally processed foods" (fresh, dried, grounded, chilled, frozen, pasteurized or fermented staple foods such as fruits, vegetables, pulses, rice, pasta, eggs, meat, fish or milk), "processed culinary ingredients" (salt, vegetable oils, butter, sugar and other substances extracted from foods and used in kitchens to transform unprocessed or minimally processed foods into culinary preparations), "processed foods" (canned vegetables with added salt, sugar-coated dry fruits, meat products only preserved by salting, cheeses and freshly made unpackaged breads, and other products manufactured with the addition of salt, sugar or other substances of the "processed culinary ingredients" group), and "ultra-processed foods" (i.e. products which undergo industrial processes notably include hydrogenation, hydrolysis, extruding, moulding, reshaping, and pre-processing by frying. Flavouring agents, colours, emulsifiers, humectants, non-sugar sweeteners and other cosmetic additives are often added to these products to imitate sensorial properties of unprocessed or minimally processed foods and their culinary preparations).

Examples of typical ultra-processed food according to the NOVA classification:

Poultry and fish nuggets and sticks and other reconstituted meat products transformed with addition of preservatives other than salt (e.g nitrites); instant noodles and dehydrated soups; carbonated drinks; sweet or savoury packaged snacks; chocolate, candies (confectionery); margarines and spreads; industrial pastries and instant desserts; breakfast 'cereals', 'energy' bars; 'energy' drinks; flavoured milk drinks; sweet desserts made from fruit with added sugars, artificial flavours and texturizing agents; cooked seasoned vegetables with ready-made sauces; vegetable patties (meat substitutes) containing food additives; meat and chicken extracts and 'instant' sauces; 'health' and 'slimming' products such as powdered or 'fortified' meal and dish substitutes; ready to heat products including pre-prepared pies, pasta and pizza dishes.

For instance, salted-only red or white meats are considered as "processed foods" whereas smoked or cured meats with added nitrites and conservatives, such as sausages and ham are classified as "ultra-processed foods". Similarly, canned salted vegetables are considered as "processed foods" whereas industrial cooked or fried seasoned vegetables, marinated in industrial sauces with added flavourings are considered as "ultra-processed foods". Regarding soups, canned liquid soups with added salts, herbs and spices are considered as "processed foods" while instant dry soup mixes are considered as "ultra-processed foods".

Example of list of ingredients for an industrial Chicken and Leek flavour soup considered as "ultra-processed" according to the NOVA classification: *"Dried Glucose Syrup, Potato Starch, Flavourings, Salt, Leek Powder (3.6%), Dried Leek (3.5%), Onion Powder, Dried Carrot, Palm Oil, Dried Chicken (0.7%), Garlic Powder, Dried Parsley, Colour [Curcumin (contains MILK)], Ground Black Pepper, MILK Protein, Stabilisers (Dipotassium Phosphate, Trisodium Citrate)".*

Distribution of food products according to NOVA categories, Open Food Facts database (n=126 556 products), France 2019

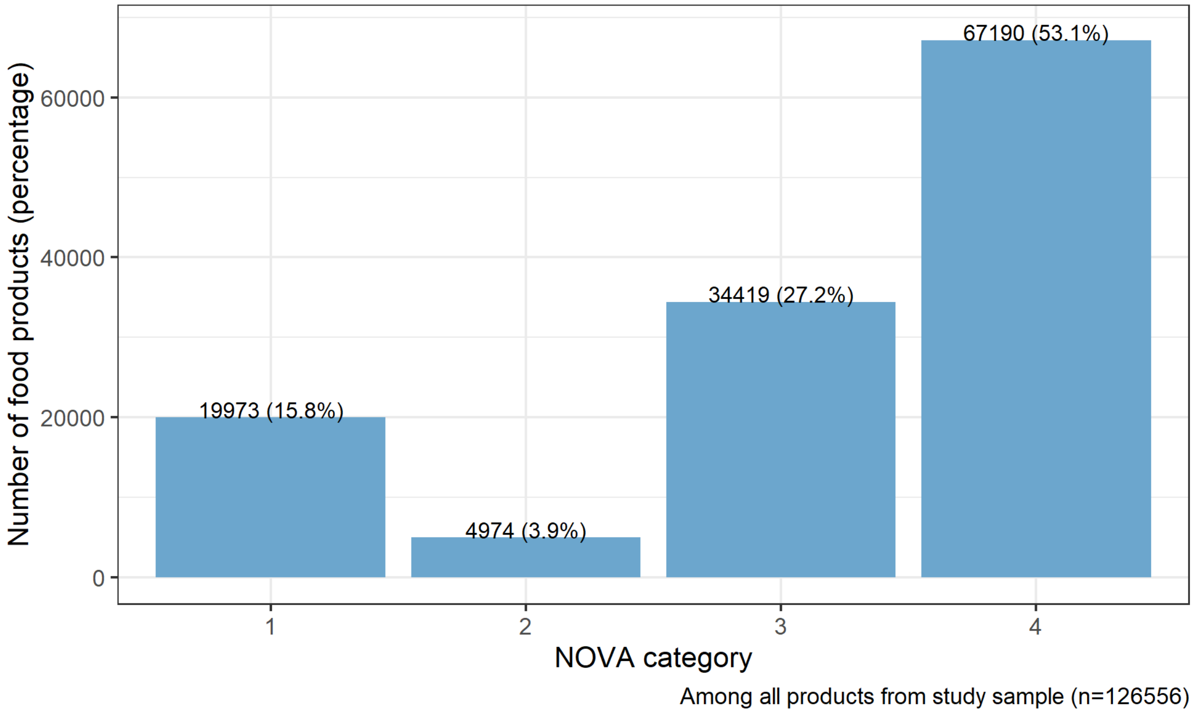

Appendix 7: Number of food products containing each food additive*, Open Food Facts database (n=126 556 products), France 2019

Number of products	Food additive
18302	E 330 Citric acid
13102	E 322 Lecithins
10100	E 14XX Modified Starches
8587	E 500 Sodium carbonates
7919	E 300 Ascorbic acid
7327	E 415 Xanthan gum
7144	E 250 Sodium nitrite
6529	E 450 Diphosphates
6504	E 440 Pectins
6060	E 471 Mono-and diglycerides of fatty acids
5535	E 412 Guar gum
5418	E 202 Potassium sorbate
5322	E 407 Carrageenan
3518	E 331 Sodium citrates
3433	E 301 Sodium ascorbate
3126	E 503 Ammonium carbonates
2839	E 316 Sodium erythorbate
2641	E 410 Locust bean gum
2462	E 422 Glycerol
2393	E 420 Sorbitols
2289	E 120 Cochineal, Carminic acid, Carmines
2274	E 270 Lactic acid
2135	E 428 Gelatine
2027	E 451 Triphosphates
2021	E 621 Monosodium glutamate
1787	E 160 Carotene
1758	E 100 Curcumin
1677	E 262 Sodium acetates
1579	E 252 Potassium nitrate
1512	E 414 Gum arabic (acacia gum)
1446	E 452 Polyphosphates
1364	E 224 Potassium metabisulphite
1283	E 950 Acesulfame K
1161	E 955 Sucralose
1136	E 296 Malic acid
1055	E 392 Extracts of rosemary
1032	E 401 Sodium alginate
1011	E 163 Anthocyanins
1008	E 282 Calcium propionate
931	E 223 Sodium metabisulphite
892	E 211 Sodium benzoate
891	E 466 Sodium carboxy methyl cellulose, Cellulose gum
887	E 326 Potassium lactate
831	E 306 Tocopherol-rich extract
828	E 341 Calcium phosphates
809	E 220 Sulphur dioxide
797	E 903 Carnauba wax
748	E 476 Polyglycerol polyricinoleate
691	E 200 Sorbic acid
688	E 133 Brilliant Blue FCF
669	E 951 Aspartame
662	E 171 Titanium dioxide
652	E 481 Sodium stearoyl-2-lactylate
599	E 325 Sodium lactate
597	E 339 Sodium phosphates
554	E 965 Maltitols
543	E 445 Glycerol esters of wood rosins
520	E 141 Copper complexes of chlorophylls, chlorophyllins
513	E 631 Disodium inosinate
508	E 162 Beetroot Red, betanin
508	E 260 Acetic acid
488	E 627 Disodium guanylate
476	E 960 Steviol glycosides
458	E 150 Caramel
453	E 1400 Dextrin
450	E 509 Calcium chloride
446	E 406 Agar
446	E 461 Methyl cellulose
440	E 290 Carbon dioxide
439	E 460 Cellulose
425	E 101 Riboflavins
413	E 334 Tartaric acid (L(+)-)
402	E 1105 Lysozyme
390	E 153 Vegetable carbon
390	E 901 Beeswax, white and yellow
387	E 304 Fatty acid esters of ascorbic acid
386	E 575 Glucono-delta-lactone
372	E 333 Calcium citrates
371	E 235 Natamycin
361	E 222 Sodium hydrogen sulphite
361	E 327 Calcium lactate
352	E 385 Calcium disodium ethylene diamine tetra-acetate (Calcium disodium EDTA)
339	E 551 Silicon dioxide
335	E 501 Potassium carbonates
334	E 170 Calcium carbonate
329	E 307 Alpha-tocopherol
329	E 340 Potassium phosphates

310	E 338 Phosphoric acid
309	E 904 Shellac
308	E 1510 Ethanon
302	E 102 Tartrazine
302	E 131 Patent Blue V
296	E 336 Potassium tartrates
287	E 524 Sodium hydroxid
274	E 502 Carbonates
261	E 172 Iron oxides and hydroxides
257	E 579 Ferrous gluconate
249	E 418 Gellan gum
248	E 920 L-cysteine
242	E 417 Tara gum
238	E 953 Isomalt
233	E 464 Hydroxypropyl methyl cellulose
231	E 473 Sucrose esters of fatty acids
209	E 1442 Hydroxy propyl distarch phosphate
205	E 320 Butylated hydroxyanisole (BHA)
204	E 635 Disodium 5'-ribonucleotides
201	E 1200 Polydextrose
197	E 475 Polyglycerol esters of fatty acids
191	E 129 Allura Red AC
187	E 508 Potassium chloride
176	E 952 Cyclamates
173	E 1422 Acetylated distarch adipate
171	E 321 Butylated hydroxytoluene (BHT)
168	E 954 Saccharins
163	E 251 Sodium nitrate
161	E 967 Xylitol
159	E 110 Sunset Yellow FCF/Orange Yellow S
153	E 1103 Invertase
152	E 140 Chlorophylls and chlorophyllins
147	E 132 Indigotine, Indigo carmine
145	E 263 Calcium acetate
144	E 350 Sodium malates
132	E 332 Potassium citrates
128	E 442 Ammonium phosphatides
125	E 1100 Alpha-Amylase
114	E 570 Fatty acids
112	E 504 Magnesium carbonates
106	E 421 Mannitol
99	E 535 Sodium ferrocyanide
93	E 127 Erythrosine
87	E 122 Azorubine, Carmoisine
85	E 492 Sorbitan tristearate
82	E 516 Calcium sulphate
79	E 572 Magnesium stearate
77	E 491 Sorbitan monostearate
76	E 124 Ponceau 4R, Cochineal Red A
76	E 1505 Triethyl citrate
74	E 261 Potassium acetates
69	E 435 Polyoxyethylene sorbitan monostearate (polysorbate 60)
69	E 968 Erythritol
63	E 444 Sucrose acetate isobutyrate
61	E 228 Potassium hydrogen sulphite
61	E 477 Propane-1,2-diol esters of fatty acids
59	E 242 Dimethyl dicarbonate
56	E 1101 Protease
56	E 640 Glycine and its sodium salt
55	E 1414 Acetylated distarch phosphate
54	E 319 Tertiary-butyl hydroquinone (TBHQ)
53	E 315 Erythorbic acid
53	E 529 Calcium oxide
51	E 142 Green S
51	E 151 Brilliant Black PN
49	E 472 Esters of mono- and diglycerides
47	E 310 Propyl gallate
47	E 433 Polyoxyethylene sorbitan monooleate (polysorbate 80)
44	E 203 Calcium sorbate
42	E 221 Sodium sulphite
41	E 210 Benzoic acid
40	E 1520 Propylene glycol
40	E 281 Sodium propionate
40	E 641 L-leucine
37	E 104 Quinoline Yellow
37	E 413 Tragacanth
37	E 525 Potassium hydroxide
37	E 536 Potassium ferrocyanide
36	E 249 Potassium nitrite
35	E 175 Gold
33	E 942 Nitrous oxide
31	E 900 Dimethyl polysiloxane
30	E 1450 Starch sodium octenyl succinate
29	E 468 Cross-linked sodium carboxy methyl cellulose, cross linked cellulose gum
29	E 482 Calcium stearoyl-2-lactylate
28	E 400 Alginic acid
27	E 1412 Distarch phosphate
26	E 425 Konjac
25	E 232 Sodium orthophenyl phenol
25	E 526 Calcium hydroxide
24	E 914 Oxidised polyethylene wax
23	E 234 Nisin
23	E 404 Calcium alginate
22	E 297 Fumaric acid
22	E 962 Salt of aspartame-acesulfame
21	E 576 Sodium gluconate
21	E 941 Nitrogen
21	E 959 Neohesperidine DC

19	E 1104 Lipase	5	E 1440 Hydroxy propyl starch
19	E 1420 Acetylated starch	5	E 231 Orthophenyl phenol
19	E 302 Calcium ascorbate	5	E 266 Sodium dehydroacetate
19	E 405 Propane-1, 2-diol alginate	5	E 411 Oat gum
19	E 510 Ammonium chloride	5	E 554 Sodium aluminium silicate
18	E 1404 Oxidised starch	5	E 650 Zinc acetate
17	E 1518 Glyceryl triacetate (triacetin)	5	E 963 Tagatose
16	E 285 Sodium tetraborate (borax)	4	E 1102 Glucose oxidase
16	E 335 Sodium tartrates	4	E 216 Propylparaben
16	E 626 Guanylic acid	4	E 236 Formic acid
15	E 212 Potassium benzoate	4	E 355 Adipic acid
14	E 103 Alkannin	4	E 505 Ammonium carbonates
14	E 155 Brown HT	4	E 522 Aluminium potassium sulphate
14	E 233 Thiabendazole	4	E 585 Ferrous lactate
13	E 182 Orcein	4	E 713 Tylosin
13	E 541 Sodium aluminium phosphate acidic	4	E 961 Neotame
13	E 552 Calcium silicate	3	E 1202 Polyvinylpolypyrrolidone
13	E 555 Potassium aluminium silicate	3	E 1204 Pullulan
12	E 1001 Choline salts	3	E 130 Indanthrene blue RS
12	E 174 Silver	3	E 1411 Distarch glycerol
12	E 363 Succinic acid	3	E 343 Magnesium phosphates
11	E 1201 Polyvinylpyrrolidone	3	E 416 Karaya gum
11	E 514 Sodium sulphates	3	E 432 Polyoxyethylene sorbitan monolaurate (polysorbate 20)
10	E 1519 Benzyl alcohol	3	E 507 Hydrochloric acid
10	E 201 Sodium sorbate	3	E 520 Aluminium sulphate
10	E 280 Propionic acid	3	E 580 Magnesium Gluconate
10	E 511 Magnesium chloride	3	E 586 4-Hexylresorcinol
10	E 530 Magnesium oxide	3	E 622 Monopotassium glutamate
9	E 402 Potassium alginate	3	E 639 Alanine
9	E 487 Sodium laurylsulphate	3	E 913 Lanolin
9	E 515 Potassium sulphates	3	E 948 Oxygen
9	E 921 L-cystine	3	E 949 Hydrogen
9	E 944 Propane	2	E 161 Xanthophylls
9	E 957 Thaumatin	2	E 181 Tannin
9	E 999 Quillaia extract	2	E 226 Calcium sulphite
8	E 1521 Polyethylene glycol	2	E 239 Hexamethylene tetramine
8	E 225 Potassium sulphite	2	E 265 Dehydroacetic acid
8	E 463 Hydroxypropyl cellulose	2	E 311 Octyl gallate
8	E 470 Salts of fatty acids	2	E 337 Sodium potassium tartrate
8	E 630 Inosinic acid	2	E 351 Potassium malate
8	E 925 Chlorine	2	E 370 1,4-Heptonolactone
7	E 329 Magnesium lactate	2	E 391 Phytic acid
7	E 958 Glycyrrhizin	2	E 431 Polyoxyethylene (40) stearate
6	E 173 Aluminium	2	E 521 Aluminium sodium sulphate
6	E 218 Methyl p-hydroxybenzoate	2	E 558 Bentonite
6	E 542 Calcium Phosphate	2	E 559 Aluminium silicate (Kaolin)
6	E 620 Glutamic acid	2	E 574 Gluconic acid
6	E 905 Microcrystalline wax	2	E 577 Potassium gluconate
6	E 966 Lactitol	2	E 578 Calcium gluconate

2	E 636 Maltol
2	E 902 Candelilla wax
1	E 106 Flavin mononucleotide
1	E 125 Ponceau SX
1	E 1410 Monostarch phosphate
1	E 143 Fast Green FCF
1	E 1503 Castor oil
1	E 152 Black 7984
1	E 180 Litholrubine BK
1	E 209 Heptylparaben
1	E 214 Ethyl-p-hydroxybenzoate
1	E 230 Biphenyl
1	E 238 Calcium formiate
1	E 284 Boric acid
1	E 303 Potassium ascorbate
1	E 312 Dodecyl gallate
1	E 315 Erythorbic acid
1	E 323 Anoxomer
1	E 328 Ammonium lactate
1	E 342 Ammonium phosphates
1	E 345 Magnesium citrate
1	E 352 Calcium malates
1	E 353 Metatartaric acid
1	E 381 Ammonium ferrocitrate
1	E 386 Disodium EDTA
1	E 419 Gum ghatti
1	E 427 Cassia gum
1	E 429 Peptone
1	E 436 Polyoxyethylene sorbitan tristearate (polysorbate 65)
1	E 443 Brominated vegetable oil
1	E 446 Succistearin
1	E 457 Alpha-Cyclodextrine
1	E 459 Beta-cyclodextrin
1	E 462 Ethyl cellulose
1	E 467 Ethulose
1	E 469 Enzymatically hydrolysed carboxy methyl cellulose, Enzymatically hydrolysed cellulose gum
1	E 486 Calcium stearoyl fumarate
1	E 493 Sorbitan monolaurate
1	E 513 Sulphuric acid
1	E 518 Magnesium sulphate
1	E 528 Magnesium hydroxide
1	E 550 Sodium silicate
1	E 553 Magnesium silicates
1	E 637 Ethyl maltol
1	E 638 Sodium aspartame
1	E 910 Wax ester
1	E 916 Calcium iodate

*** decreasing number of occurrences. Number of products containing each of the specific modified starches may be underestimated in the database due to lack of detail on food packaging**

Appendix 8a: Stability plot of food additive clusters determined by the ClustOfVar R package, Open Food Facts database, France 2019

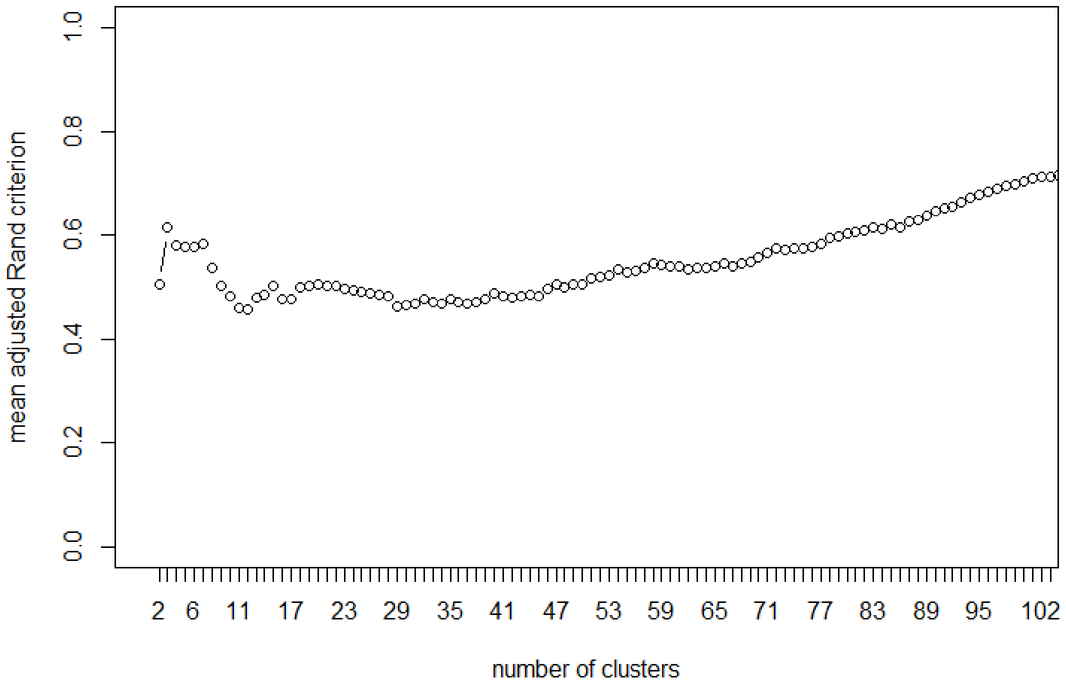

Appendix 8b: Cluster of food additives and respective squared loadings determined by the ClustOfVar R package, Open Food Facts database, France 2019

Cluster	Squared loading	Food additive
Cluster 1: dyes and glazing agents mostly used in sweets (n=24 food additives)	0.471	E 903 Carnauba wax
	0.288	E 901 Beeswax, white and yellow
	0.274	E 171 Titanium dioxide
	0.236	E 100 Curcumin
	0.203	E 133 Brilliant Blue FCF
	0.17	E 131 Patent Blue V
	0.168	E 414 Gum arabic (acacia gum)
	0.167	E 120 Cochineal, Carminic acid, Carmines
	0.165	E 163 Anthocyanins
	0.146	E 350 Sodium malates
	0.115	E 296 Malic acid
	0.11	E 904 Shellac
	0.1	E 153 Vegetable carbon
	0.086	E 132 Indigotine, Indigo carmine
	0.073	E 141 Copper complexes of chlorophylls, chlorophyllins
	0.065	E 161b Lutein
	0.065	E 172 Iron oxides and hydroxides
	0.064	E 162 Beetroot Red, betanin
	0.05	E 102 Tartrazine
	0.044	E 110 Sunset Yellow FCF/Orange Yellow S
	0.04	E 160c Paprika extract, capsanthin, capsorubin
	0.04	E 129 Allura Red AC
	0.015	E 140 Chlorophylls and chlorophyllins
	0.011	E 445 Glycerol esters of wood rosins
Cluster 2: wide range of additives mostly used in sandwiches and sugary desserts (n=61 food additives)	0.262	E 415 Xanthan gum
	0.254	E 14xx Modified Starches
	0.254	E 471 Mono-and diglycerides of fatty acids
	0.233	E 412 Guar gum
	0.178	E 407 Carrageenan
	0.177	E 330 Citric acid
	0.163	E 202 Potassium sorbate
	0.12	E 410 Locust bean gum
	0.106	E 160a Carotenes
	0.097	E 331 Sodium citrates
	0.079	E 401 Sodium alginate
	0.077	E 300 Ascorbic acid
	0.07	E 282 Calcium propionate
	0.057	E 224 Potassium metabisulphite
	0.055	E 472e Mono- and diacetyl tartaric acid esters of mono- and diglycerides of fatty acids
	0.054	E 472b Lactic acid esters of mono- and diglycerides of fatty acids
	0.053	E 160 Carotene
	0.05	E 440 Pectins
	0.049	E 481 Sodium stearoyl-2-lactylate
	0.048	E 270 Lactic acid
	0.044	E 466 Sodium carboxy methyl cellulose, Cellulose gum
	0.033	E 428 Gelatine
	0.024	E 417 Tara gum
	0.023	E 475 Polyglycerol esters of fatty acids
	0.022	E 339 Sodium phosphates
	0.02	E 101 Riboflavins
	0.02	E 200 Sorbic acid
	0.016	E 150a Plain caramel
	0.016	E 211 Sodium benzoate

	0.015	E 332 Potassium citrates
	0.015	E 333 Calcium citrates
	0.014	E 460 Cellulose
	0.014	E 575 Glucono-delta-lactone
	0.013	E 385 Calcium disodium ethylene diamine tetra-acetate (Calcium disodium EDTA)
	0.013	E 260 Acetic acid
	0.013	E 509 Calcium chloride
	0.012	E 341 Calcium phosphates
	0.012	E 160b Annatto, Bixin, Norbixin
	0.011	E 461 Methyl cellulose
	0.01	E 222 Sodium hydrogen sulphite
	0.009	E 1422 Acetylated distarch adipate
	0.008	E 920 L-cysteine
	0.008	E 472c Citric acid esters of mono- and diglycerides of fatty acids
	0.008	E 263 Calcium acetate
	0.007	E 223 Sodium metabisulphite
	0.006	E 1442 Hydroxy propyl distarch phosphate
	0.005	E 327 Calcium lactate
	0.004	E 150b Caustic sulphite caramel
	0.004	E 464 Hydroxypropyl methyl cellulose
	0.004	E 220 Sulphur dioxide
	0.002	E 392 Extracts of rosemary
	0.002	E 406 Agar
	0.001	E 1510 Ethanon
	0.001	E 1105 Lysozyme
	0.001	E 579 Ferrous gluconate
	0.001	E 1100 Alpha-Amylase
	0.001	E 418 Gellan gum
	0.001	E 504 Magnesium carbonates
	0	E 570 Fatty acids
	0	E 251 Sodium nitrate
	0	E 235 Natamycin
Cluster 3: stabilizers and emulsifiers mostly used in biscuits and cakes (n=13 food additives)	0.64	E 500 Sodium carbonates
	0.431	E 450 Diphosphates
	0.29	E 322 Lecithins
	0.281	E 503 Ammonium carbonates
	0.263	E 422 Glycerol
	0.196	E 420 Sorbitols
	0.036	E 476 Polyglycerol polyricinoleate
	0.033	E 473 Sucrose esters of fatty acids
	0.028	E 336 Potassium tartrates
	0.024	E 334 Tartaric acid (L(+)-)
	0.009	E 1103 Invertase
	0.003	E 442 Ammonium phosphatides
	0.002	E 524 Sodium hydroxid
Cluster 4: sweeteners mostly used in sugar-free chewing gums and artificially sweetened	0.486	E 950 Acesulfame K
	0.48	E 951 Aspartame
	0.36	E 967 Xylitol
	0.348	E 965 Maltitols
	0.341	E 421 Mannitol
	0.279	E 321 Butylated hydroxytoluene (BHT)

beverages (n=19 food additives)	0.218	E 953 Isomalt
	0.217	E 955 Sucralose
	0.111	E 320 Butylated hydroxyanisole (BHA)
	0.056	E 170 Calcium carbonate
	0.04	E 338 Phosphoric acid
	0.032	E 952 Cyclamates
	0.025	E 1200 Polydextrose
	0.023	E 954 Saccharins
	0.022	E 150d Sulphite ammonia caramel
	0.007	E 960 Steviol glycosides
	0.004	E 1400 Dextrin
	0.002	E 290 Carbon dioxide
	0	E 150 Caramel
Cluster 5: flavor enhancers additives mostly used in instant noodles and other umami-tasting foods (n=13 food additives)	0.781	E 631 Disodium inosinate
	0.771	E 627 Disodium guanylate
	0.448	E 621 Monosodium glutamate
	0.096	E 551 Silicon dioxide
	0.085	E 501 Potassium carbonates
	0.074	E 635 Disodium 5'-ribonucleotides
	0.048	E 150c Ammonia caramel
	0.032	E 306 Tocopherol-rich extract
	0.009	E 340 Potassium phosphates
	0.003	E 304 Fatty acid esters of ascorbic acid
	0.001	E 307 Alpha-tocopherol
	0	E 502 Carbonates
	0	E 307c Tocopherol
Cluster 6: preservatives and antioxidants mostly used in processed meat (n=11 food additives)	0.633	E 250 Sodium nitrite
	0.383	E 316 Sodium erythorbate
	0.362	E 301 Sodium ascorbate
	0.272	E 451 Triphosphates
	0.233	E 262 Sodium acetates
	0.198	E 326 Potassium lactate
	0.104	E 325 Sodium lactate
	0.104	E 452 Polyphosphates
	0.086	E 252 Potassium nitrate
	0.051	E 407a Processed euchema seaweed
	0.015	E 508 Potassium chloride

REFERENCES

1. Chavent, M., Kuentz-Simonet, V., Liquet, B. & Saracco, J. ClustOfVar: An R Package for the Clustering of Variables. *Journal of Statistical Software* **50**, 1–16 (2012).
2. Chavent, M., Vanessa, K. & Saracco, J. Orthogonal rotation in PCAMIX. *Adv Data Anal Classif* **6**, 131–146 (2012).
3. van Borkulo, C. D. *et al.* A new method for constructing networks from binary data. *Sci Rep* **4**, 5918 (2014).
4. Speed, T. P. & Kiiveri, H. T. Gaussian Markov Distributions over Finite Graphs. *The Annals of Statistics* **14**, 138–150 (1986).
5. Chen, J. & Chen, Z. Extended Bayesian information criteria for model selection with large model spaces. *Biometrika* **95**, 759–771 (2008).
6. Julia, C., Etilé, F. & Hercberg, S. Front-of-pack Nutri-Score labelling in France: an evidence-based policy. *The Lancet Public Health* **3**, e164 (2018).
7. Julia, C. *et al.* Application of the British Food Standards Agency nutrient profiling system in a French food composition database. *Br.J Nutr.* **112**, 1699–1705 (2014).
8. Egnell, M., Hercberg, S. & Julia, C. Development and validation of the Nutri-Score A colour-coded summary front-of-pack nutrition label. *eFOOD Lab_International* (2019).
9. Monteiro, C. A. *et al.* NOVA. The star shines bright. *1* **7**, 28–38 (2016).
10. Monteiro, C. A. *et al.* The UN Decade of Nutrition, the NOVA food classification and the trouble with ultra-processing. *Public Health Nutr.* 1–13 (2017) doi:10.1017/S1368980017000234.
11. Moubarac, J.-C., Parra, D. C., Cannon, G. & Monteiro, C. A. Food Classification Systems Based on Food Processing: Significance and Implications for Policies and Actions: A Systematic Literature Review and Assessment. *Curr Obes Rep* **3**, 256–272 (2014).
